# Co-transcriptional genome surveillance by HUSH is coupled to termination machinery

**DOI:** 10.1016/j.molcel.2023.04.014

**Published:** 2023-05-09

**Authors:** Andrew L. Spencley, Shiran Bar, Tomek Swigut, Ryan A. Flynn, Cameron H. Lee, Liang-Fu Chen, Michael C. Bassik, Joanna Wysocka

**Affiliations:** 1 Department of Chemical and Systems Biology, Stanford University School of Medicine, Stanford, CA, USA; 2 Cancer Biology Program, Stanford University School of Medicine, Stanford, CA, USA; 3 Stem Cell Program, Boston Children’s Hospital, Boston, MA, USA; 4 Department of Stem Cell and Regenerative Biology, Harvard University, Cambridge, MA, USA; 5 Department of Genetics, Stanford University School of Medicine, Stanford, CA, USA; 6 Department of Developmental Biology, Stanford University School of Medicine, Stanford, CA, USA; 7 Institute of Stem Cell Biology and Regenerative Medicine, Stanford University School of Medicine, Stanford, CA, USA; 8 Howard Hughes Medical Institute, Stanford University School of Medicine, Stanford, CA, USA; 9 Lead contact

## Abstract

The HUSH complex recognizes and silences foreign DNA such as viruses, transposons, and transgenes without prior exposure to its targets. Here, we show that endogenous targets of the HUSH complex fall into two distinct classes based on the presence or absence of H3K9me3. These classes are further distinguished by their transposon content and differential response to the loss of HUSH. A *de novo* genomic rearrangement at the *Sox2* locus induces a switch from H3K9me3-independent to H3K9me3-associated HUSH targeting, resulting in silencing. We further demonstrate that HUSH interacts with the termination factor WDR82 and—via its component MPP8—with nascent RNA. HUSH accumulates at sites of high RNAPII occupancy including long exons and transcription termination sites in a manner dependent on WDR82 and CPSF. Together, our results uncover the functional diversity of HUSH targets and show that this vertebrate-specific complex exploits evolutionarily ancient transcription termination machinery for co-transcriptional chromatin targeting and genome surveillance.

## In brief

We show that the HUSH complex accumulates at sites of high RNAPII occupancy in a manner dependent on termination factors WDR82 and CPSF. However, co-transcriptional genome surveillance by HUSH can be decoupled from silencing. Two classes of HUSH targets are distinguished by the presence or absence of H3K9me3 and transposon content.

## Graphical Abstract

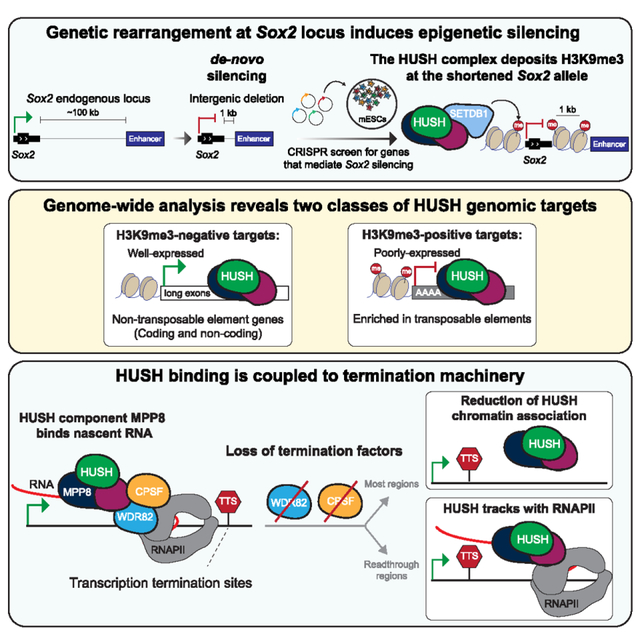

## INTRODUCTION

Invasion by foreign DNA, such as viruses and transposons, poses an ever-present threat to genome integrity against which all species must defend themselves. Hence, diverse mechanisms for restricting mobile element activity have evolved across eukaryotes (reviewed in Almeida et al.^1^). Repression of such foreign elements is mediated by the deposition of repressive histone modifications (such as trimethylation of histone H3 lysine 9 [H3K9me3]) and DNA methylation marks.^2–5^ Heterochromatin formation is often guided toward regions recognized as “non-self” DNA via sequence-dependent mechanisms involving small RNAs or DNA-binding proteins.^3,5–10^ For example, in mammalian cells, the Krüppel-associated box zinc finger protein (KRAB-ZFP) family has evolved to recognize specific DNA sequences for binding transposable elements (TEs). KRAB-ZFPs recruit epigenetic silencing machinery including TRIM28 and the histone methyltransferase SETDB1, which subsequently deposits H3K9me3.^10,11^ In germ cells, the PIWI-interacting RNA (piRNA) system utilizes small RNA guides to identify expressed TEs by forming double-stranded RNAs.^6–9^ Although these systems employ distinct mechanisms of TE targeting, both rely on the recognition of sequence-specific features, thereby requiring memory of non-self elements that are developed over an evolutionary timescale. In stark contrast, the vertebrate-specific human silencing hub (HUSH) complex was recently found to repress a diverse set of foreign DNA targets without previously encountering their sequences.^12^

The core HUSH members MPP8, TASOR (FAM208A), and PPHLN1 (periphilin) were initially identified as regulators of transgene silencing in mammalian cells.^13^ The HUSH complex binds to chromatin and recruits the ATP-dependent chromatin remodeler MORC2 and methyltransferase SETDB1 to deposit H3K9me3 and silence its targets. Since its discovery, the list of HUSH-silenced targets has expanded to include LINE-1 retrotransposons (L1s), endogenous retroviruses (ERVs), the HIV provirus, and various transgenes with diverse sequence features.^12–18^ However, the molecular mechanism by which HUSH identifies these broad classes of non-self-targets remains elusive.

Despite its well-characterized role as a silencing complex, there is mounting evidence that HUSH silencing is coupled to sites of active transcription.^12,15^ We have previously demonstrated that HUSH binds and represses euchromatic L1s and that induction of transcription through L1 leads to HUSH accumulation.^15^ Intriguingly, the presence of introns protects HUSH-targeted transgenes from H3K9me3 silencing.^12^ Furthermore, MPP8 has been shown to interact with ZCCHC8 to promote RNA degradation of TE targets through the nuclear exosome targeting (NEXT) complex.^14^ Collectively, these observations suggest a link between HUSH and the co-transcriptional processes.

Here, we find that the deletion of a gene desert separating the highly expressed *Sox2* gene and its super-enhancer induces transcriptional silencing, which we subsequently show through a genetic screen to be HUSH-dependent. These observations coupled with genome-wide analyses reveal that the endogenous HUSH targets fall into two distinct classes. The first class of HUSH targets is poorly expressed, enriched in H3K9me3 and evolutionarily young TEs, and is transcriptionally silenced by HUSH. In contrast, the second and more abundant class of HUSH targets is devoid of H3K9me3 and transposons and instead contains expressed coding and non-coding genes. A gene desert deletion at the *Sox2* locus induces a switch from H3K9me3-independent to H3K9me3-associated HUSH binding, resulting in HUSH-mediated silencing. We further demonstrate that HUSH accumulates at sites of high RNAPII occupancy, especially at long exons and transcription termination sites (TTS). HUSH binding is dependent on the termination factors WDR82 and cleavage and polyadenylation specificity factor (CPSF). Together, our results reveal the functional diversity of HUSH targets, uncover a role for the termination machinery in HUSH targeting to chromatin, and suggest that target recognition by HUSH is a dynamic, co-transcriptional process.

## RESULTS

### Intragenic deletion yields unexpected epigenetic silencing at *Sox2* locus

**Figure 1.**
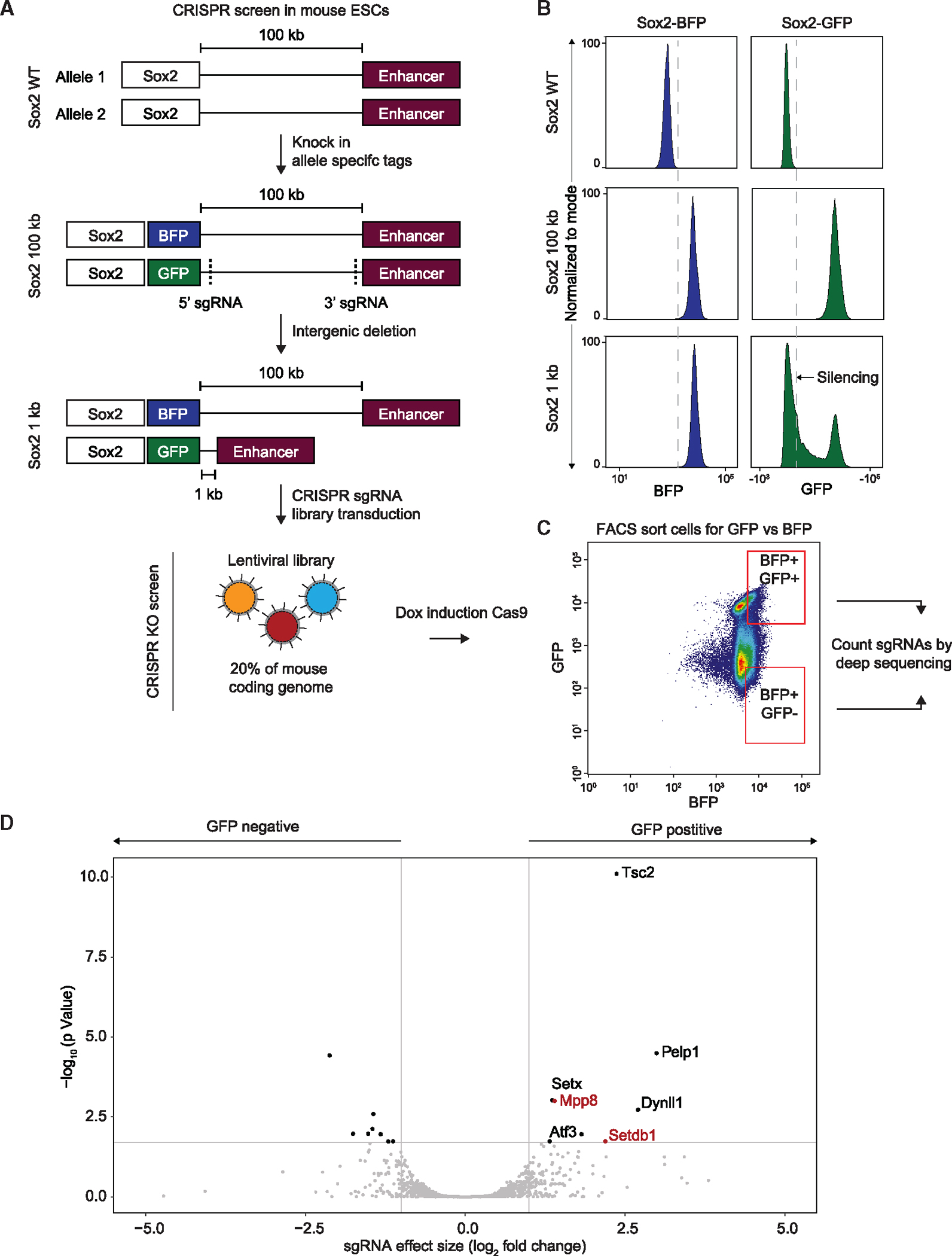
Intragenic deletion results in unexpected epigenetic silencing at the *Sox2* locus (A) Schematic diagram of the *Sox2* locus and its associated CRISPR-Cas9 screen in mESCs: in WT mESCs, *Sox2*, and its super-enhancer are separated by a 100 kb gene desert (top). Allele-specific GFP and BFP tags were integrated into the *Sox2* gene (middle) and two sgRNA were used to delete the space between *Sox2* and its enhancer (bottom). CRISPR screen was conducted by transducing a library of sgRNAs covering 20% of the mouse coding genome and induction of Cas9 expression (bottom). (B) Fluorescence-activated cell sorting (FACS) histogram plots of BFP (left) and GFP (right) in WT (top), *Sox2*-*100kb* (center), and *Sox2*-*1kb* (bottom) mESCs. (C) FACS 2D plots of BFP (x axis) and GFP (y axis) in Sox2–1kb mESCs following transduction with sgRNA library covering 20% of the mouse coding genes and after induction of Cas9 expression. Marked in red is the gating strategy that was used to sort BFP+/GFP+ and BFP+/GFP− populations, which was followed by deep-sequencing and differential analysis of sgRNAs enrichment. (D) Differential enrichment of sgRNAs between GFP-positive and GFP-negative populations that were sorted in (C) as calculated by DE-Seq. Marked in red aregenes associated with the HUSH complex.

We initially developed a system to probe the effects of genomic distance on enhancer activity, generating an endogenous *Sox2* reporter in mouse embryonic stem cells (mESCs) by knocking in allele-specific GFP or BFP tags at the C terminus (Figure 1A). Next, uniquely on the *Sox2-GFP* allele, we shortened the distance between the well-characterized *Sox2* super enhancer^19^ located 100 kb downstream of the gene to 1 kb while leaving the *Sox2-BFP* allele intact (Figure 1A; hereafter we will refer to the shortened *Sox2-GFP* allele as *Sox2–1kb*). Given recent findings that enhancer activity can decay with distance,^20^ we expected that the expression from the *Sox2–1kb* allele would increase. To our surprise, although expression from the full-length *Sox2-BFP* allele remained unchanged, the shortened *Sox2–1kb* allele was silenced, as measured by BFP and GFP fluorescence, respectively (Figure 1B). Single-molecule RNA FISH with GFP- or BFP-specific probes confirmed that this silencing occurred at the RNA level (Figure S1A).

To investigate why the *Sox2–1kb* allele was silenced despite the deletion of a transcriptionally inert gene desert, we designed a CRISPR knockout (KO) screen to identify potential silencing mediators. Doxycycline-inducible Cas9 and lentiviral sgRNA libraries were integrated into the parental *Sox2–1kb* line (Figures 1A and S1B). Due to limitations in throughput associated with a fluorescence-activated cell sorting (FACS)-based screen, we selected two sub-library pools covering 20% of the mouse coding genome that included the highest number of nuclear-localized targets. BFP-positive cells enriched or depleted for GFP, corresponding to populations in which silencing was lost or maintained, respectively, were then sorted by FACS and guide RNAs analyzed by sequencing (Figure 1C). We discovered guides for multiple genes significantly enriched in the GFP-positive vs. negative cells, indicating that loss of these genes might rescue the expression from the silenced allele (Figure 1D; Tables S1 and S2). Importantly, two of these genes, *Mpp8* and *Setdb1*, are both components of the HUSH complex,^13^ suggesting a role for HUSH in silencing the short *Sox2–1kb* allele (Figure 1D).

### The HUSH complex is responsible for silencing the *Sox2* short allele

**Figure 2.**
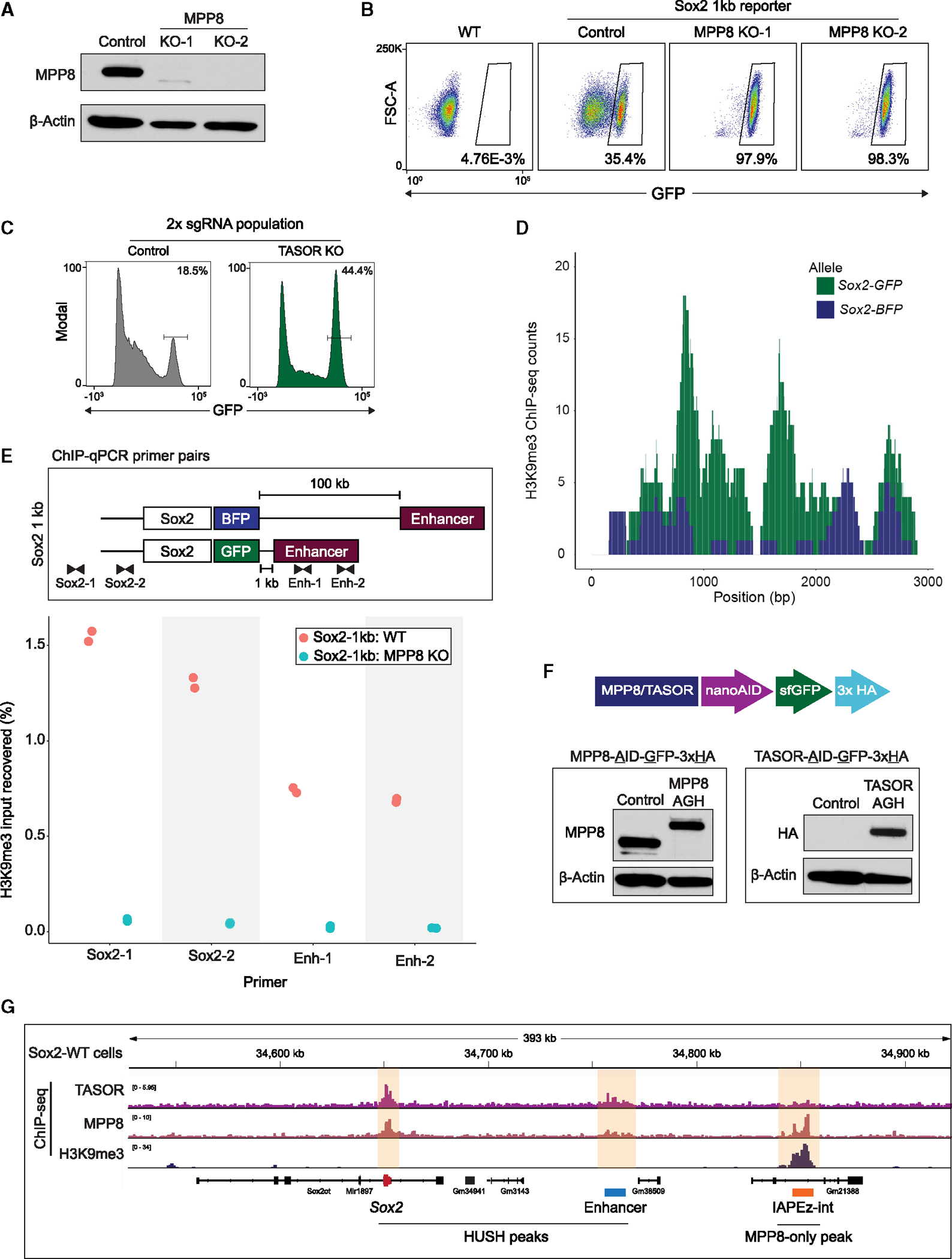
The HUSH complex is responsible for silencing at the *Sox2* short allele (A) MPP8 western blot in Sox2–1kb reporter (control) and MPP8 knockout (KO) subclones. Membranes were probed with antibodies against MPP8 and β-actin as a loading control. (B) FACS 2D plots of GFP (x axis) and forward scatter (y axis) in WT mESCs, Sox2–1kb reporter (control), and MPP8 KO clones. (C) FACS histogram plots of GFP in *Sox2–1kb* reporter cells transduced with two non-targeting sgRNAs (control) or TASOR-targeting sgRNAs (TASOR KO), 9 days after induction of Cas9 expression. (D) H3K9me3 ChIP-seq coverage aligned independently to *Sox2-GFP* (green) or *Sox2-BFP* (blue) alleles in the *Sox2–1kb* cell line. (E) Chromatin immunoprecipitation quantitative PCR (ChIP-qPCR) of H3K9me3 in Sox2–1kb reporter. Schematic diagram of the Sox2 locus in the Sox2–1kb reporter and location of primers used for the qPCR (top). Percentage of input recovered for H3K9me3 ChIP in Sox2–1kb reporter control (red) and MPP8 KO clones (blue) with the different primers for qPCR (bottom). (F) Top: schematic diagram of the MPP8 and TASOR endogenous tagging strategy in WT mESCs (termed “AGH” for AID-GFP-HAx3). Bottom: western blots in WT (control) and endogenously tagged mESCs, probing for MPP8 (left), HA (right) and β-actin as a loading control. (G) Browser tracks of TASOR, MPP8 and H3K9me3 ChIP-seq in WT mESCs surrounding the *Sox2* locus. Highlighted in yellow are the major ChIP-seq pileups for either MPP8 and TASOR (HUSH peaks) or MPP8-only peaks.

To confirm the involvement of the HUSH complex in silencing the *Sox2–1kb* reporter, we generated clonal MPP8 KO mESC lines in the *Sox2–1kb* background (Figure 2A). Loss of MPP8 rescued the observed silencing phenotype and restored GFP expression (Figure 2B). Furthermore, KO of TASOR, a defining subunit of HUSH, also dramatically increased the proportion of GFP-positive cells in the population of *Sox2–1kb* mESCs transduced with TASOR-targeting sgRNAs (Figure 2C). Notably, TASOR-targeting sgRNAs were not present in the CRISPR library used in our screen (which only covered 20% of the genome), and thus, TASOR was not previously detected as a hit in this screen. The HUSH complex recruits SETDB1 to deposit the histone modification H3K9me3 and repress its targets^13,21^; we assessed the enrichment of H3K9me3 by chromatin immunoprecipitation quantitative PCR (ChIP-qPCR) and sequencing (ChIP-seq) in the *Sox2–1kb* mESCs. We observed the accumulation of H3K9me3 at both *Sox2* and its enhancer, which was specific to the GFP allele (Figure 2D). KO of MPP8 significantly relieved H3K9me3 (Figure 2E), confirming that HUSH targets the *de novo* genomic rearrangement at the *Sox2* locus, resulting in the silencing of the gene and its enhancer.

Previous reports suggested structural similarities between HUSH and the RNA-induced transcriptional silencing (RITS) complex in fission yeast.^22^ Silencing by the RITS complex is dependent on the RNAi machinery component Dicer.^23^ To test if this also applies to HUSH, we used CRISPR-Cas9 to KO Dicer1 in the *Sox2–1kb* reporter line (Figures S1C and S1D). In contrast to the significant increase in GFP expression upon the loss of HUSH (Figures 2B and 2C), Dicer deletion did not rescue the silencing of the GFP allele (Figure S1E), thereby demonstrating that—unlike the RITS complex—HUSH silencing is independent of RNAi.

Although many seemingly diverse HUSH targets have been identified previously, they are predominantly viral and TE-derived sequences or exogenous transgenes.^15,16,18,22,24,25^ Furthermore, much of our current understanding of HUSH biology is based on transgenic reporter assays.^18,24,26^ Upon validation of HUSH as our silencing culprit, we sought to understand this *de novo* target in a native context, both prior to and after the genomic rearrangement. For this purpose, we endogenously tagged the C terminus of both TASOR and MPP8 with an auxin-inducible degron (AID) as well as GFP and HA tags (hereafter, AGH), allowing us to both rapidly deplete the proteins with piggyBac-integrated TIR1 and circumvent known issues with TASOR endogenous antibodies (Figure 2F). We performed ChIP-seq for TASOR-AGH, MPP8, and H3K9me3 in wild-type (WT) mESCs, initially focusing on the *Sox2* locus (Figure 2G). Surprisingly, even in unperturbed conditions, we observed robust binding of MPP8 and TASOR to both *Sox2* and its distal enhancer. However, consistent with the abundant expression of *Sox2* in mESCs, there was no apparent H3K9me3 at either of these sites in WT cells. Of note, we observed a downstream intracisternal A-type particle (IAP) element tiled heavily with H3K9me3 and bound by MPP8, but not by TASOR (Figure 2G). However, the H3K9me3 did not spread beyond this IAP element in the *Sox2–1kb* cells and was not affected by the MPP8 KO (Figure S1F).

Given the function of HUSH as an H3K9me3-dependent silencing complex, the presence of MPP8 and TASOR at the highly transcribed *Sox2* locus seemed counterintuitive. Combined with our observation that the shortened *Sox2–1kb* allele is silenced by HUSH and H3K9me3, these observations suggest the existence of distinct modes of HUSH association with chromatin.

### Two classes of HUSH targets distinguished by H3K9me3, transposon content, transcriptional activity, and response to HUSH loss

**Figure 3.**
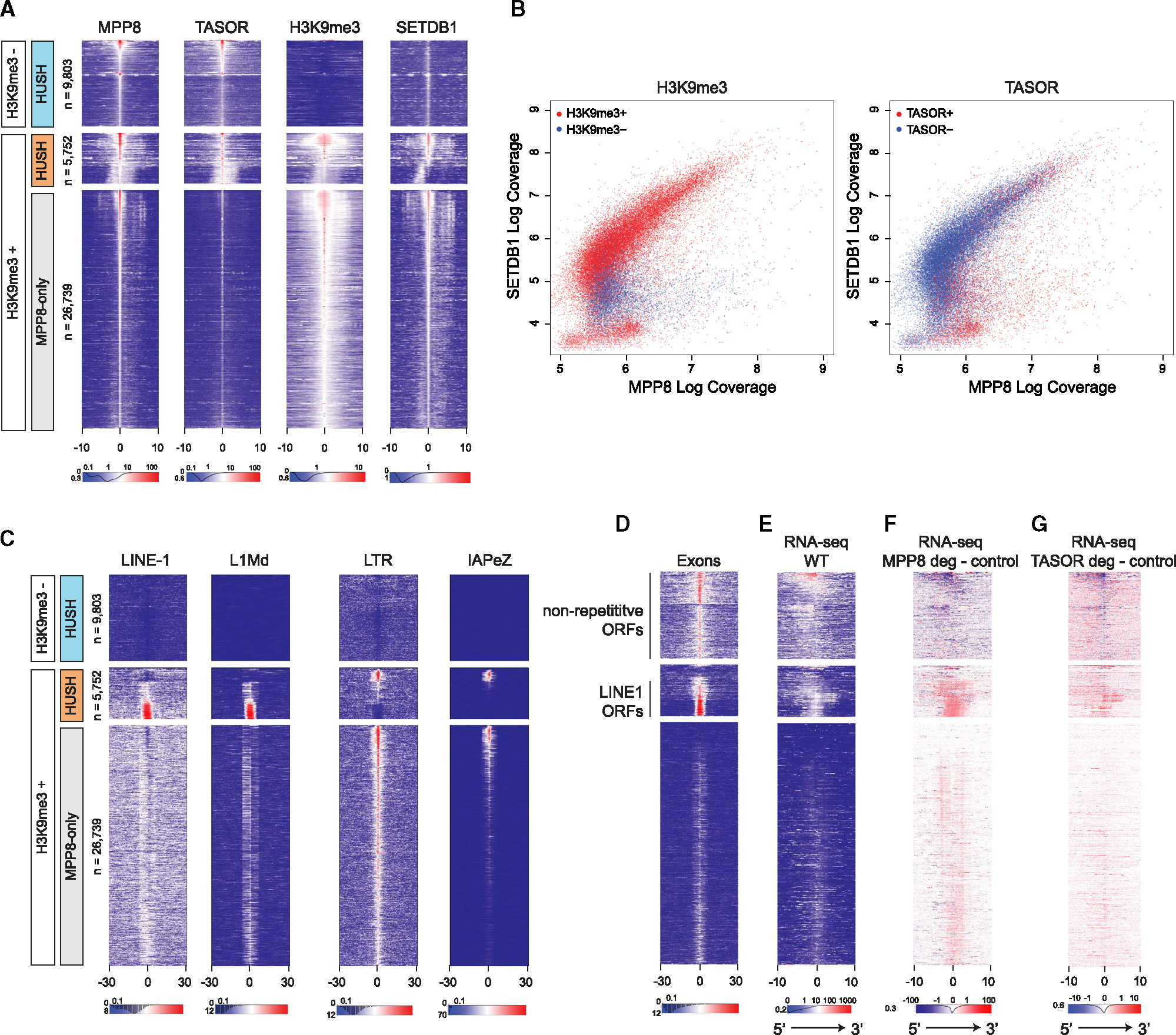
Two classes of HUSH targets distinguished by H3K9me3, transposon content, and response to HUSH depletion (A) Heatmaps showing ChIP-seq coverage for MPP8, TASOR, H3K9me3, and SETDB1 at MPP8 peaks. The x axis represents distance from MPP8 ChIP peak in kb. Heatmaps were sorted first by H3K9me3 and then by MPP8 and TASOR signal. Regions with high coverage of both MPP8 and TASOR were classified as HUSH targets (H3K9me3-positive; orange and H3K9me3-negative; light blue), whereas regions with low or no TASOR were delineated as MPP8-only (gray). The number of peaks for each class (n) is indicated. ChIP peak orientation in heatmaps is stranded based on the ratio of positive- and negative-stranded PRO-seq signals. (B) Scatterplots showing all MPP8 and TASOR ChIP-seq peaks as a function of log coverage for MPP8 (x axis) and SETDB1 (y axis) ChIP-seq. Marked in red are peaks overlapping H3K9me3 (left) or TASOR (right). (C) Heatmaps showing the presence and relative position of repetitive elements as annotated by repeat masker, centered and sorted as in (A). The x axis represents distance from MPP8 ChIP peak in kb. (D) Heatmaps showing relative position of transcribed exons as annotated by custom transcriptome models derived from RNA-Seq of WT mESCs, centered and sorted as in (A). The x axis represents distance from MPP8 ChIP peak in kb. (E) Heatmaps showing RNA-seq coverage in WT mESCs, centered and sorted as in (A). The x axis represents distance from MPP8 ChIP peak in kb. (F) Heatmaps showing subtraction of RNA-seq coverage in MPP8-depleted mESCs (treated with auxin for 7 days) from control mESCs, centered and sorted as in (A). The x axis represents distance from MPP8 ChIP peak in kb. (G) Heatmaps showing subtraction of RNA-seq coverage in TASOR-depleted mESCs (treated with auxin for 1 day) from untreated control mESCs, centered and sorted as in (A). The x axis represents distance from MPP8 ChIP peak in kb.

With the surprising discovery of HUSH binding (but not silencing) the WT *Sox2* locus, we used our H3K9me3, TASOR-AGH, and MPP8 ChIP-seq data to explore whether other endogenous HUSH targets without H3K9me3 exist. Consistent with the reported functions of MPP8 in additional protein complexes beyond HUSH,^27–29^ we called more peaks for MPP8 (n = 26,739) than for TASOR (n = 15,555) (Figure 3A). To uncouple HUSH binding from H3K9me3 and from HUSH-independent MPP8 peaks, we first binned MPP8 ChIP-seq peaks into H3K9me3-positive and -negative regions. We then distinguished bona fide HUSH targets by the enrichment of TASOR (Figure 3A). This analysis allowed us to identify and visualize three classes of genomic targets: HUSH without H3K9me3, HUSH + H3K9me3, and MPP8-only sites, representing HUSH-independent binding of MPP8 to chromatin (Figure 3A). Strikingly, despite the known role of HUSH as a silencing complex, most HUSH peaks (9,803 of the 15,555 total TASOR peaks) are found in regions devoid of H3K9me3, similar to what we observed at the WT *Sox2* locus. Re-analysis of published SETDB1 ChIP-seq^30^ revealed that HUSH sites with H3K9me3 and MPP8-only sites are both enriched for SETDB1 (Figure 3B), with a high correlation between MPP8 and SETDB1 binding. In contrast, HUSH targets without H3K9me3 display weak SETDB1 binding (Figures 3A and 3B), suggesting that SETDB1 is preferentially recruited to (or stabilized at) a select subset of HUSH sites, subsequently resulting in their methylation at H3K9.

Given that the majority of previously identified endogenous HUSH targets are evolutionarily young TEs,^15^ we intersected our data with positions of known repetitive element families to identify their distribution within the three above-described classes (Figure 3C). Consistent with known HUSH targets,^15,16^ evolutionary young L1 elements such as L1Md along with long terminal repeats (LTRs) such as IAPEz dominated the H3K9me3-positive HUSH regions but interestingly had unique and well-defined signatures (Figure 3D). Specifically, full-length L1s were mostly targeted by HUSH but not by MPP8 alone and displayed relatively lower levels of H3K9me3. In contrast, LTRs had overall higher levels of H3K9me3 and were bound either by the HUSH complex or MPP8 alone. Within the HUSH-bound regions, the LTR targets primarily corresponded to active IAPEz-int LTRs, whereas the MPP8-only regions harbored additional LTR subfamilies, consistent with broad targeting of LTRs by the MPP8-containing TRIM28 complex^11,16^ (Figures 3C and S2A).

In contrast to the H3K9me3-enriched regions, the H3K9me3-negative HUSH targets were devoid of L1 and LTR elements (Figure 3C). We systematically compared the enrichment of all annotated repeat classes at H3K9me3-positive vs. negative HUSH sites. As expected, H3K9me3-positive sites were enriched in young L1 and LTR retrotransposon families, including L1Md and various IAP and MERV-K elements (Figure S2A). In contrast, H3K9me3-negative HUSH targets were largely devoid of TEs and instead enriched in tRNA genes and tRNA-derived repeats (Figure S2A). Further analysis of trinucleotide repeats revealed selective enrichment of A-trinucleotides at the H3K9me3-positive sites, especially pronounced at L1s, in agreement with their known A-skew (Figure S2B). Thus, in addition to SETDB1 binding, TE enrichment and A-richness further distinguish H3K9me3-positive and -negative HUSH targets.

We next focused on additional genomic features that may characterize distinct HUSH targets. We found that both H3K9me3-positive and -negative HUSH targets, but not MPP8-only sites, are enriched in exons (Figure 3D). Among the H3K9me3-positive HUSH targets, the exon signals mostly corresponded to L1 ORFs, although dense HUSH binding was also associated with large clusters of repetitive KZFP genes, harboring H3K9me3 at their 3′ ends (Figure S2F). H3K9me3-negative regions were associated with both non-coding and protein-coding genes. The gene ontology analysis at these regions showed enrichment in genes associated with chromatin organization and nucleosome assembly (Figure S2C). This is driven at least in part by the histone genes, as exemplified by strong HUSH binding at the tight array of abundantly expressed single-exon histone genes (Figure S2G). In contrast, we failed to detect any significant gene ontology categories enriched at the H3K9me3-positive sites.

Given that HUSH silencing has been reported to be transcription-dependent,^12,15^ we analyzed the total RNA levels at HUSH and MPP8-only targets by rRNA-depleted RNA-seq. In contrast to the MPP8-only sites, we observed detectable RNA expression at all HUSH targets. However, consistent with their repression by HUSH, the H3K9me3-positive sites showed substantially lower transcriptional activity compared with sites devoid of H3K9me3 (Figure 3E). We next asked how the loss of HUSH affects RNA levels at these two target classes. We utilized our endogenously tagged MPP8-AGH and TASOR-AGH cell lines to acutely deplete MPP8 and TASOR, respectively (Figure S2D), and examined total RNA expression (Figures 3F and 3G). As expected, H3K9me3-positive HUSH targets were de-repressed upon MPP8 or TASOR depletion. Surprisingly, however, the H3K9me3-negative HUSH targets showed decreased RNA expression in the absence of MPP8 or TASOR (Figures 3F and 3G). Thus, the two classes of targets identified in our study respond distinctly to the loss of HUSH. Of note, at the long TASOR depletion time points (10 days), the transcription from the H3K9me3-negative targets was upregulated in the auxin-treated cells relative to the untreated control (Figure S2E), suggesting a compensatory mechanism upon prolonged loss of HUSH. Altogether, our analyses identified two classes of HUSH targets that are distinguished by their epigenetic status, TE content, and differential response to HUSH perturbation.

### The HUSH complex interacts with RNA polymerase II-associated factors and binds nascent RNA

**Figure 4.**
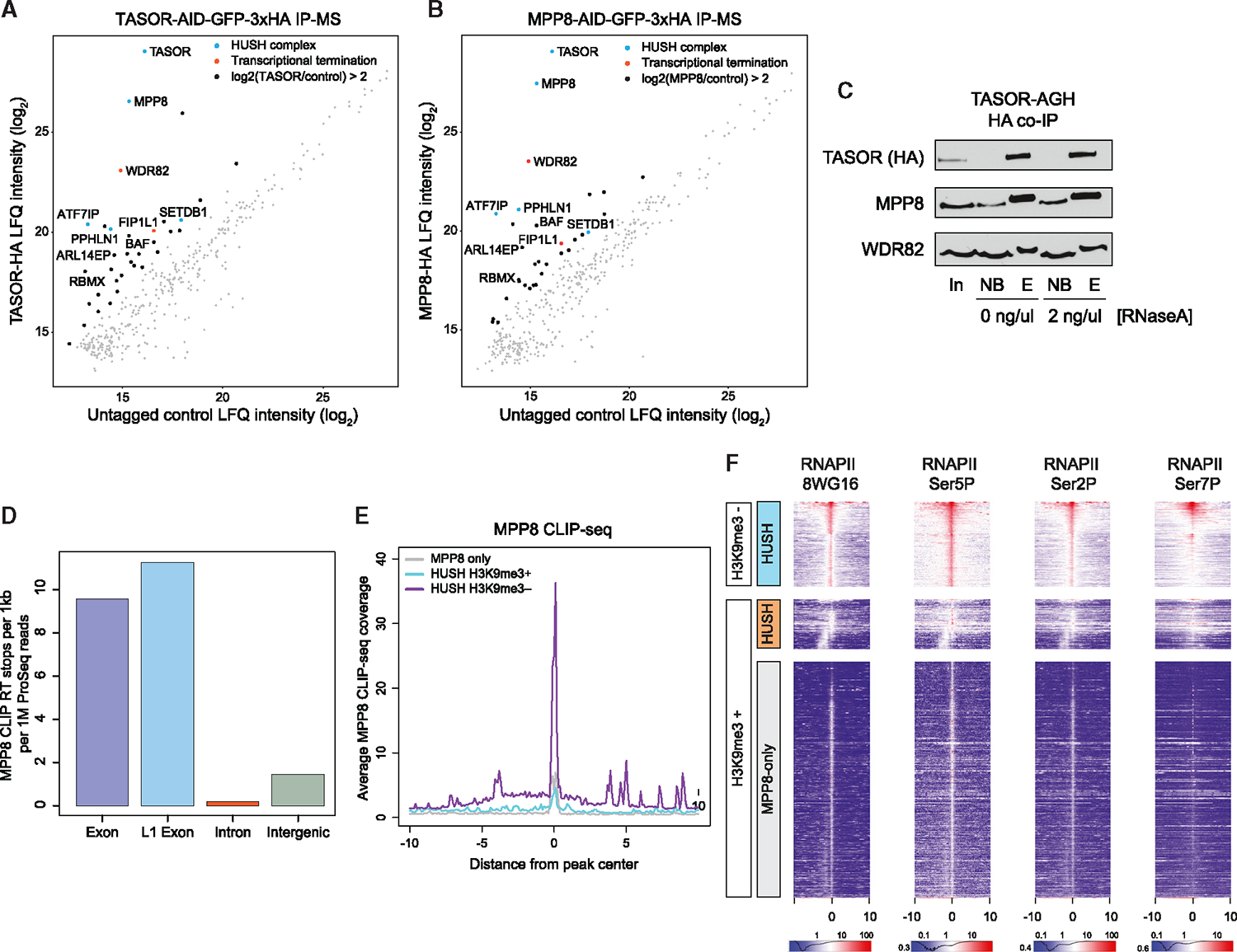
The HUSH complex interacts with RNA polymerase II termination factors and binds nascent RNA (A) Log of label-free quantification (LFQ) intensity for HA immunoprecipitation (IP) followed by mass-spectrometry in TASOR-AGH (y axis) and untagged control mESCs (x axis). Colored dots highlight associations of proteins with the HUSH complex (blue) or with RNA polymerase II (red). (B) Same as (A), for MPP8-AGH IP-MS. (C) Western blot showing coIP of MPP8 and WDR82 with TASOR (HA IP) in the TASOR-AGH cell line. Samples were incubated with RNaseA at 2 ng/μL during the IP. Inputs (in), non-bound flowthrough (NB), and HA peptide eluted (E) fractions at shown. (D) Enrichment of MPP8 irCLIP-seq for various genomic features. y axis was calculated by normalizing the number of RT stops to both length and level of transcription, as measured by PRO-seq. Exons were annotated by custom transcriptome models derived from RNA-seq of WT mESCs. (E) Aggregate plot showing MPP8 CLIP-seq normalized read count (y axis) relative to the distance from HUSH or MPP8-only ChIP peaks (x axis). HUSH peaks were divided into H3K9me3-positive (blue), H3K9me3-negative (purple), or MPP8-only regions based on the classification in Figure 3A. (F) Heatmaps showing ChIP-seq coverage for RNA polymerase II using different antibodies that recognize various C-terminal domain (CTD) modifications, centered and sorted as in Figure 3A. The x axis represents distance from MPP8 ChIP peak in kb.

To identify candidate molecular players that may mediate the function and targeting of HUSH at these distinct classes of sites, we utilized the MPP8-AGH and TASOR-AGH cell lines to perform immunoprecipitation followed by mass spectrometry (IP-MS) (Figures 4A and 4B; Table S3). Overall, and despite MPP8 participating in other molecular complexes, we detected striking similarities between MPP8 and TASOR interactors, including PPHLN1, the third member of the HUSH complex, and the known protein partners SETDB1 and ATF7IP. Interestingly, we identified FIP1L1, a member of the CPSF complex, as an interactor of both MPP8 and TASOR. Furthermore, we identified WDR82, another regulator of transcription termination and a direct RNAPII C-terminal domain (CTD)-binding protein,^31,32^ to be enriched in our IPs to a comparable extent as canonical HUSH members (Figures 4A and 4B). To validate WDR82 as a putative HUSH interactor, we pulled down TASOR in our TASOR-AGH cell line and indeed retrieved both MPP8 and WDR82 by coIP; this interaction was not disrupted with RNAseA treatment (Figure 4C). Together, our results uncover interactions of HUSH with the RNAPII-associated transcription termination factors.

We next asked if HUSH binds nascent RNA, like many components of the RNA processing machinery. Previous studies showed that HUSH indeed binds RNA through PPHLN1^12^ and structural analyses suggested RNA binding for TASOR.^22^ To characterize HUSH RNA binding patterns genome-wide, we utilized infrared UV-C crosslinking IP sequencing (irCLIP-seq). We first tested MPP8 and TASOR for their capacity to directly bind RNA by irCLIP-PAGE and determined MPP8 to be the most robust (although this does not preclude a role for other HUSH components in RNA binding; Figures S3A and S3B). We proceeded to extract the crosslinked RNA species for sequencing and mapped RT stops, representing the nucleotide at which MPP8 is directly crosslinked to RNA, and observed strong binding to RNA derived from diverse genomic features, with the highest percentage of RT-stops mapping within introns, consistent with nascent RNA binding (Figure S3C). The irCLIP signal could not simply be explained by correlation with RNA abundance, as many transcripts expressed at relatively low levels were bound by MPP8 (Figure S3D). Furthermore, when we normalized to both feature length and transcript level, it was evident that MPP8 predominantly bound exonic RNAs from both genic and L1 regions (Figure 4D), reminiscent of the enrichment of exons at HUSH genomic occupancy sites (Figure 3D). We observed strong enrichment of MPP8 irCLIP reads at the H3K9me3-negative HUSH ChIP peaks but not at the MPP8-only sites, suggesting that despite MPP8 participating in multiple protein complexes, the observed CLIP signals can be largely attributed to HUSH-associated RNAs (Figures 4E, S3E, and S3F).

The observation that the HUSH complex interacts with nascent RNA stemming from the sites of its chromatin occupancy and binds transcription termination machinery suggests that it may be tracking with the RNAPII. In agreement, when we reanalyzed previously published RNAPII ChIP-seq datasets, including those for distinct CTD phosphorylation states,^33^ we observed enrichment of RNAPII at HUSH peaks, especially pronounced at H3K9me3-negative sites (Figures 4F and S4A). Together, our results are consistent with the model whereby HUSH associates with RNAPII-bound processing factors and with nascent RNA and accumulates at the sites of high RNAPII occupancy.

### WDR82 regulates HUSH accumulation on chromatin

**Figure 5.**
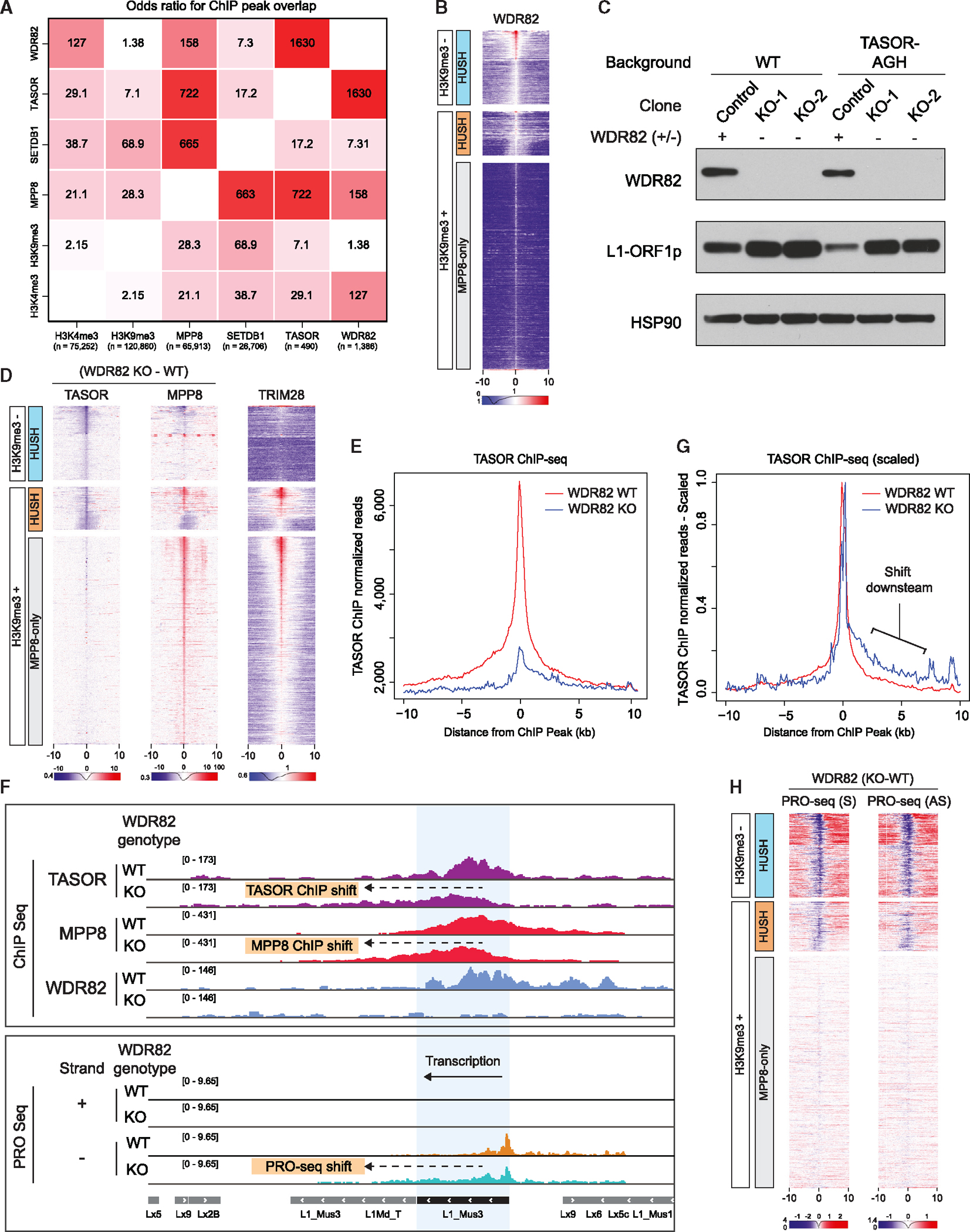
WDR82 is required for HUSH accumulation on chromatin (A) Heatmap showing odds ratio values for overlap between H3K4me3, H3K9me3, MPP8, TASOR, and WDR82 ChIP-seq peaks. Peaks were called using MACS2 with broad peak settings. Statistics were calculated by BEDTools fisher. The number of peaks for respective proteins is indicated at the bottom. (B) Heatmaps of WDR82 ChIP-seq coverage in WT mESCs, centered and sorted as in Figure 3A. The x axis represents distance from MPP8 ChIP peak in kb. (C) Western blot of WT and TASOR-AGH mESC total cell lysates. Control and WDR82 KO clones from each cell line are shown. Membranes were probed with antibodies against WDR82, L1-ORF1p, and HSP90 as a loading control. (D) Heatmaps showing TASOR (left) or MPP8 (middle) differential ChIP-seq coverage in WDR82 KO – WT backgrounds compared with reanalyzed ChIP-seq coverage for TRIM28 (right). Signal is centered and sorted as in Figure 3A. The x axis represents distance from MPP8 ChIP peak in kb. (E) Aggregate plot showing TASOR ChIP-seq coverage (y axis) relative to the distance from HUSH ChIP peak (x axis) in WT (red) and WDR82 KO (blue) mESCs. (F) Top: browser tracks of TASOR, MPP8 and WDR82 ChIP-seq in WT and WDR82 KO mESCs. Bottom: browser tracks of PRO-seq in WT (orange) and WDR82 KO (blue) mESCs for the positive (+) and negative (−) DNA strands. The arrow marks the observed shifts in ChIP-seq and PRO-seq signals in WDR82 KO samples. (G) Aggregate plot showing TASOR ChIP-seq coverage individually scaled to span (0,1) interval in each sample (y axis) to visualize changes in plot shape, relative to the distance from HUSH ChIP peak (x axis) in WT (red) and WDR82 KO (blue) mESCs. (H) Heatmaps showing subtraction of PRO-seq coverage (WDR82 KO – WT), for sense (S, left) and antisense (AS, right) strands. Heatmaps were centered and sorted as in Figure 3A. The x axis represents distance from MPP8 ChIP peak in kb.

We next examined the mechanism by which HUSH is recruited to the RNAPII machinery. We focused on WDR82, which was an attractive candidate for multiple reasons: (1) it is the most highly enriched HUSH interactor in our IP-MS experiments (Figures 4A and 4B) and (2) it directly associates with and regulates the RNAPII CTD, playing roles in the recruitment of the Set1 H3K4me3 methyltransferase complex to RNAPII and in transcriptional termination.^31,32,34–36^ We performed ChIP-seq for WDR82 and H3K4me3 and compared WDR82 genomic occupancy with that of TASOR, MPP8, SETDB1, H3K9me3, and H3K4me3 by calculating odds ratios for ChIP-seq peak overlap (Figure 5A). As expected, the binding of TASOR and MPP8 was highly overlapping (odds ratio 722). Furthermore, WDR82 was correlated with H3K4me3, consistent with its role in Set1 methyltransferase complexes (odds ratio 127).^37^ Strikingly, the strongest odds ratio among all pairwise overlaps was that of WDR82 and TASOR (1,630; Figure 5A). Further analysis revealed that WDR82 was enriched at both H3K9me3-negative and H3K9me3-positive targets, with more robust binding at the H3K9me3-negative regions, whereas the MPP8-only sites were not bound by WDR82 (Figures 5B and S4B).

To test the effects of WDR82 on HUSH genomic occupancy, we generated clonal WDR82 KO mESC lines in both WT and TASOR-AGH genetic backgrounds and validated the loss of WDR82 in these cells by immunoblotting (Figure 5C). Loss of WDR82 did not affect the stability of the HUSH complex components or the interaction between TASOR and MPP8 (Figure S4C). Strikingly, however, TASOR binding was drastically diminished at both H3K9me3-positive and -negative sites (Figures 5D, 5E). Furthermore, loss of WDR82 was associated with diminished MPP8 binding at HUSH-targeted L1 elements and H3K9me3-negative HUSH sites (Figure 5D). In contrast, MPP8 signals increased at HUSH-bound IAPeZ elements and MPP8-only sites, most of which are LTR retrotransposons, broadly targeted by the MPP8-containing KZFP-TRIM28 complexes (Figures 3C, 5D, and S2A). Indeed, when we reanalyzed existing TRIM28 mESC ChIP-seq data and juxtaposed them against the effects of WDR82 KO on MPP8 binding,^16^ we observed that MPP8 sites upregulated in WDR82 KO cells corresponded to the TRIM28-bound sites (Figure 5D). We speculate that this effect is due to competition for MPP8 between HUSH and KZFP-TRIM28 complexes: once HUSH is depleted from chromatin, more MPP8 may be available to associate with the TRIM28 complex. Regardless, this result demonstrates the specificity of our observations—it is indeed uniquely at HUSH-only sites that MPP8 binding to chromatin is diminished by the loss of WDR82. WDR82 KO was also accompanied by the upregulation of L1 ORF1p protein (Figure 5C), indicating the importance of WDR82 for HUSH-dependent L1 silencing.

Upon examination of genome browser tracks of ChIP-seq signals in WDR82 KO cells, we noticed that although at most targets HUSH binding was lost, at a smaller subset of loci, TASOR remained bound to chromatin, but the peak was shifted relative to its position in WT cells (Figures 5F and S4D). Indeed, although the overall TASOR ChIP-seq signal was greatly diminished in WDR82 KO cells (Figure 5E), it also became asymmetric, extending further downstream in the sense direction (Figure 5E; comparison of scaled aggregate plots is shown in Figure 5G). Loss of WDR82 was previously shown to result in transcriptional readthrough caused by defects in RNAPII termination.^31^ To test if transcriptional readthrough contributed to the observed shifts in HUSH binding, we used precision run-on sequencing (PRO-seq) to identify changes in nascent transcription in WDR82 KO cells. Consistent with RNAPII ChIP-seq, we observed high PRO-seq signals at HUSH-bound regions, but not at MPP8-only sites (Figure S4E). WDR82 KO resulted in a significant reduction in PRO-seq signals at HUSH targets (Figure 5H). At the sites characterized by downstream shifts in HUSH binding, we observed diminished PRO-seq signals at the center of the original peak and readthrough transcription coincident with the direction of the HUSH ChIP-seq shift (Figures 5F and S4D). Consistent with previous observations that WDR82 preferentially affects the termination of non-coding transcripts, many of the sites with transcriptional readthrough and accompanied reduction or shift in HUSH binding were long non-coding RNA genes such as *Malat1, Neat1*, and *Tsix* (Figures S5A–S5C). Collectively, our data show that WDR82 significantly affects the stable association of HUSH with chromatin and reveals concordant changes in HUSH ChIP-seq and PRO-seq upon loss of WDR82.

### Silencing at the *Sox2–1kb* locus is WDR82-dependent and associated with perturbation of termination signals

We next returned to the *Sox2* locus to understand what signals may cause the switch between the H3K9me3-negative and -positive HUSH binding. As expected, given the global effect of WDR82 depletion on HUSH binding, we observed that both TASOR and MPP8 binding at the *Sox2* locus were dramatically reduced in WDR82 KO cells (Figure S6A, top panel). Furthermore, loss of WDR82 was associated with transcriptional readthrough at the *Sox2* gene (Figure S6A, middle panel, top PRO-seq tracks on the sense strand) and enhancer (Figure S6A, PRO-seq on the sense/antisense strands). To test if WDR82 loss is required for HUSH-mediated silencing of the *Sox2–1kb* allele, we stably integrated doxycycline-inducible Cas9 and two sgRNAs for WDR82, TASOR, or negative control regions, and monitored silencing of Sox2-GFP by FACS over time (Figure S6B). Although the *Sox2–1kb* allele remained silenced in the negative control cells, KO of either TASOR or WDR82 rescued GFP expression to a comparable extent (Figure S6B).

We then performed PRO-seq in the *Sox2–1kb* reporter cells and compared nascent transcription in these cells with that of WT cells, with a caveat that the nascent RNA signal comes both from the shortened GFP allele and full-length BFP allele. As expected, given the HUSH-dependent silencing of the *Sox2–1kb* allele, the loss of MPP8 enhanced PRO-seq signal at the locus (Figure S6A). Additionally, we observed that the genomic rearrangement resulted in the loss of the native TTS, and this was accompanied by spurious transcription on the sense strand downstream of deletion directly adjacent to the enhancer, suggesting that the *Sox2–1kb* intergenic deletion generated an ectopic TTS (Figure S6A). To directly test whether perturbation of the termination signal can drive the silencing of the shortened *Sox2* allele, we generated a 590 bp heterozygous deletion spanning termination-associated elements at the *Sox2*-*GFP* allele in the full-length *Sox2–100kb* reporter line (Figure S6C); this TTS deletion resulted in significant loss of the GFP signal in a subpopulation of cells and in the accumulation of H3K9me3 at the locus (Figures S6D and S6E).

### H3K9me3-negative HUSH targets overlap transcription termination sites

**Figure 6.**
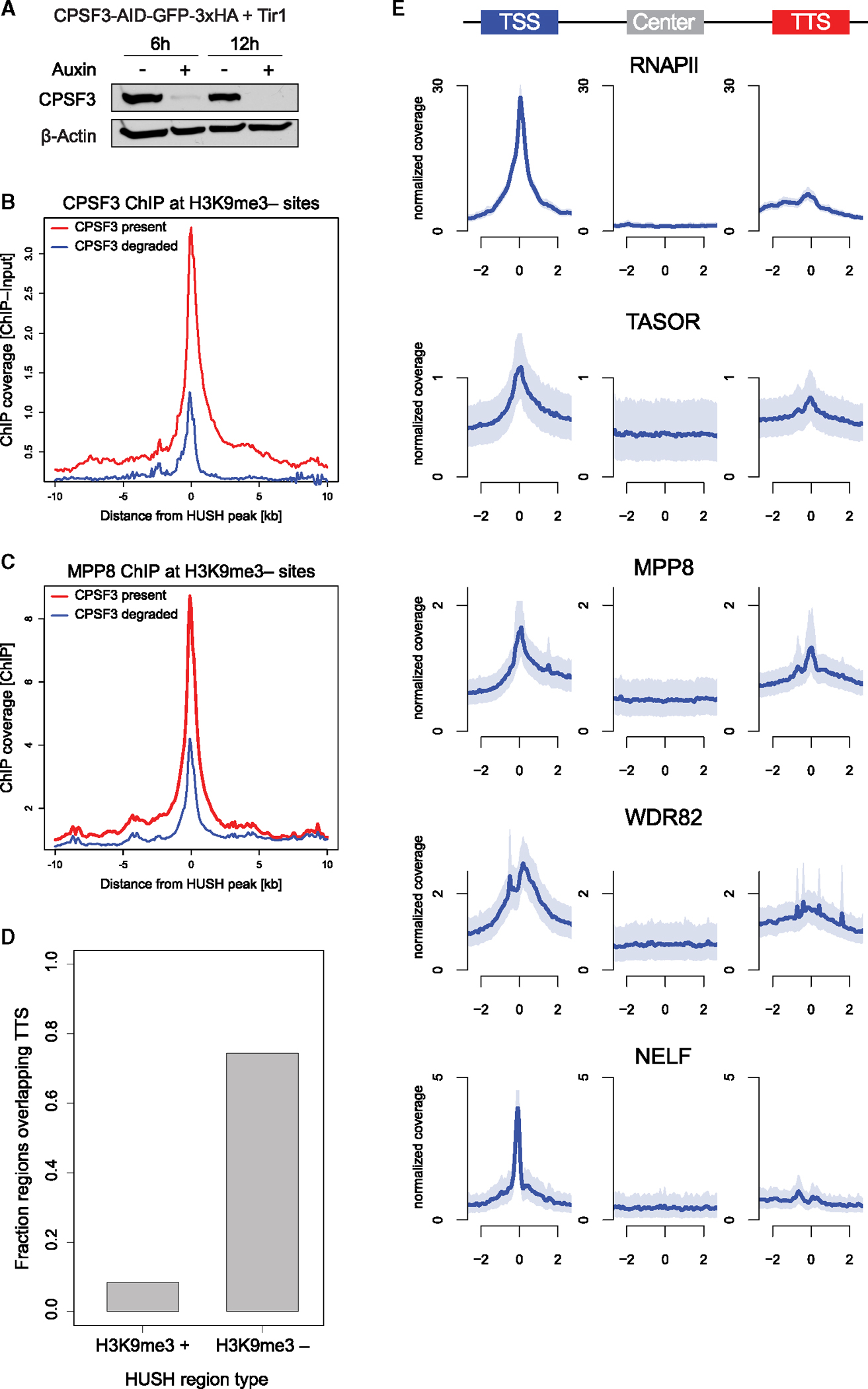
HUSH is coupled to transcription termination machinery (A) Western blot in CPSF3-AGH mESCs, either with or without auxin treatment for 6 or 12 hours. Membranes were probed with antibodies against CPSF3 and β-actin. (B) Average plot of CPSF3 ChIP-seq coverage at H3K9me3-negative HUSH peaks in CPSF3-AGH cells with or without auxin represented by blue or red lines, respectively. The x axis indicates distance from HUSH ChIP peak in kb. (C) Average plot of MPP8 ChIP-seq coverage at H3K9me3-negative HUSH peaks in CPSF3-AGH cells with or without auxin represented by blue or red lines, respectively. The x axis indicates distance from HUSH ChIP peak in kb. (D) Fraction of HUSH peaks that overlap GENCODE-annotated transcription termination sites (TTS) for H3K9me3-positive and H3K9me3-negative regions. (E) Aggregate plots of TASOR, MPP8, WDR82, RNAPII (8WG16), and NELF ChIP-seq coverage centered on unique TSS, midpoints, or TTS (based on polyA) for H3K9me3-negative HUSH sites. Plotted are the median (solid line) and 95% CI of 1,000× bootstrap. The ordinate represents genomic distance relative to the feature in kb and the abscissa represents coverage (reads per billion per base per region).

Our observations suggest links between HUSH targeting to chromatin and transcription termination machinery. We noted that another termination factor, the CPSF complex member FIP1L1, was among the most enriched HUSH-associated proteins (Figures 4A and 4B). To test if CPSF also contributes to HUSH targeting, we used endogenous targeting to tag its core subunit CPSF3 with AID, which on auxin addition resulted in acute loss of CPSF3 and termination defects (Figures 6A and S7A). Subsequent analysis of CPSF3 and MPP8 binding by ChIP-seq revealed accumulation of CPSF3 at H3K9me3-negative HUSH targets and a decrease in MPP8 occupancy at these sites following acute CPSF3 degradation (Figures 6B, 6C, S7D, and S7E).

Strong enrichment of WDR82 and CPSF3 at H3K9me3-negative HUSH sites prompted us to examine their overlap with TTS. Indeed, this analysis revealed that nearly 75% of H3K9me3-negative HUSH targets overlap an annotated TTS (Figure 6D). We note, however, that our analysis of the H3K9me3-positive HUSH sites likely underestimates their overlap with terminators due to poor TTS annotation in TEs and the presence of many cryptic TTS in such elements. Given that H3K9me3-negative HUSH sites overlap regions where various forms of RNAPII accumulate and that WDR82 has been previously linked to H3K4me3, a mark enriched at promoters and 5′ gene ends,^38,39^ we analyzed HUSH enrichment at TSS, gene bodies, and TTS.

We compared ChIP-seq coverage of TASOR, MPP8, and WDR82 to that previously published for RNAPII and NELF.^33,40^ We observed that—similarly to RNAPII—TASOR, MPP8, and WDR82 are enriched at both the TSS and TTS of H3K9me3-negative HUSH targets, whereas NELF signal is only detected at TSS, as expected (Figure 6E). However, the relative ratio of TASOR, MPP8, and WDR82 to RNAPII is higher at TTS than at TSS, suggesting that HUSH preferentially interacts with RNAPII accumulating at terminators. Finally, although HUSH binds at a subset of TTS in a termination machinery-dependent manner, we did not observe transcriptional readthrough at these sites on MPP8 KO, whereas such readthrough was evident upon loss of CPSF or WDR82 (Figures S5C, S6B, and S7A–S7C). Furthermore, the overall PRO-seq signal at the HUSH-bound TTS did not diminish in the absence of MPP8, in contrast to what we observed for WDR82 (Figures S5C, S7B, and S7C). These observations suggest that HUSH targeting is coupled to but not per se required for transcription termination.

## DISCUSSION

The HUSH complex has emerged as a central mechanism for protecting vertebrate genomes against the invasion of active transposons, retroviruses, and transgenes due to its unique ability to detect and repress mobile elements without prior exposure to their sequences.^12,13,15,16,18^ Recent efforts have expanded the determinants of HUSH targeting to include active transcription, lack of introns, overall length, and skewed nucleotide content.^12,15^ However, a mechanistic understanding of how HUSH is recruited to its chromatin targets is still lacking.

**Figure 7.**
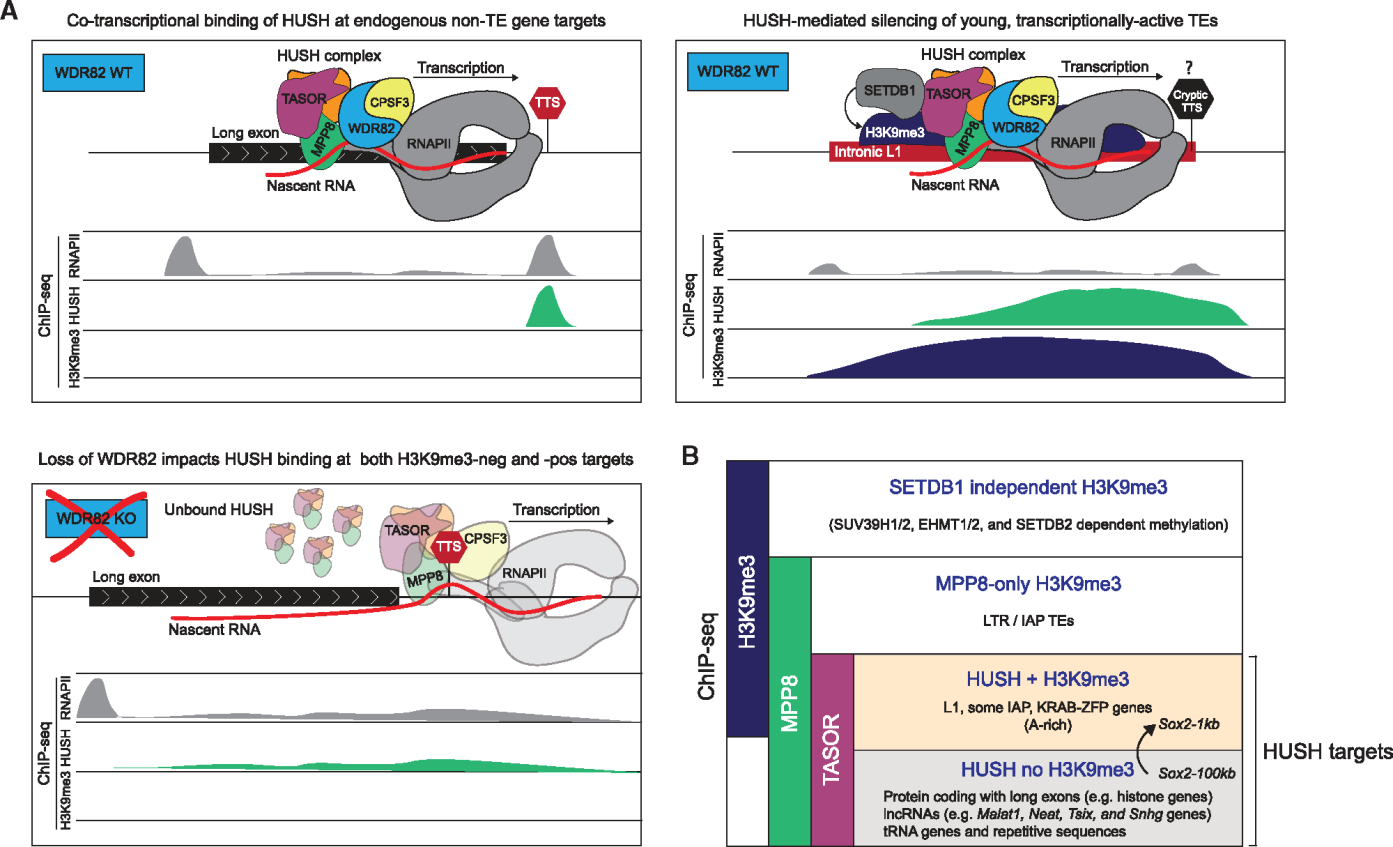
Model for co-transcriptional, termination machinery-coupled targeting of HUSH to endogenous genic and transposon targets (A) Schematic model for the mechanism of HUSH binding to endogenous, non-TE gene targets absent H3K9me3 (top-left), and silencing of young, transcriptionally-active TEs (top-right). HUSH associates with termination factors WDR82 and CPSF and tracks with RNAPII to survey transcribed portion of the genome. Loss of WDR82 results in transcriptional readthrough by RNAPII and prevents stable interaction of HUSH with chromatin at both H3K9me3-positive and -negative HUSH targets. We hypothesize that recognition of a cryptic TTS in an L1 or at the *Sox2–1kb* allele may provide a trigger H3K9me3. (B) Summary of the different regions and relevant features that are associated with H3K9me3 and/or HUSH ChIP-seq signals.

Here, we made a serendipitous discovery that deletion of a gene desert separating the highly expressed *Sox2* gene and its super-enhancer induces HUSH-dependent silencing. Both the gene and the super-enhancer are bound by HUSH prior to the genomic rearrangement and transcriptional repression, which ultimately led us to the realization that the majority of sites targeted by HUSH in mESCs are uncoupled from silencing and H3K9me3. These H3K9me3-negative sites are distinct from the canonical HUSH targets in that they exhibit low levels of SETDB1 binding, are devoid of TEs, and are enriched for transcribed exons and TTS. In agreement with the close link between HUSH binding and transcription termination, we found that termination factors WDR82 and CPSF interact with HUSH and regulate its chromatin occupancy. These results, together with previously published observations, allow us to propose a mechanistic model of co-transcriptional genome surveillance by HUSH (Figure 7A). In this model, actively transcribed regions of the genome are dynamically monitored by HUSH through a mechanism involving interactions between HUSH and RNAPII-associated termination machinery (Figure 7A).

In agreement, we and others demonstrated that HUSH associates with co-transcriptional RNA processing factors and nascent RNA and that its targeting to chromatin is dependent on active transcription^12,15,24^ and termination factors WDR82 and CPSF3 (this study). At genomic sites where HUSH binding is incompletely lost upon WDR82 KO, HUSH shifts in the direction of the RNAPII transcriptional readthrough (Figure 7A). Throughout the genome, HUSH accumulates at sites where RNAPII elongation is known to slow down, such as long exons and transcription termination zones. Notably, the PNUTS-PP1 complex is recruited by WDR82 to directly regulate RNAPII CTD and induce stalling at termination sites.^35^

Another important discovery of a co-transcriptional process that affects HUSH function has come from a recent study demonstrating that inclusion of introns in long cDNA transgenes can counteract HUSH-mediated repression.^12^ Since invading mobile elements are often products of reverse transcription and thus are intronless, such a strategy could distinguish invading retroelements from host genes. Given a documented role of splicing machinery in counteracting premature transcript cleavage and polyadenylation,^41,42^ and conversely, the preferential role of WDR82 in the termination of poorly spliced transcripts, we speculate that opposing effects of introns and WDR82 on HUSH recruitment may reflect active competition between splicing and termination. Furthermore, introns prevent the usage of strong polyadenylation site (PAS) terminators, and it has been recently hypothesized that this occurs through conformational changes in RNAPII that hinder the recruitment of termination factors like WDR82.^43^ Such a model could explain the observation that introns protect against HUSH-mediated silencing.

HUSH-dependent silencing occurs at a subset of targets recognized as non-self, such as evolutionary young L1s, LTR retrotransposons, or invading retroviruses (Figure 7B). Our finding that a gene desert deletion at the *Sox2* locus induces a switch from the H3K9me3-independent to H3K9me3-associated HUSH binding suggests that HUSH is continuously monitoring the transcribed genome to deploy silencing when the non-self DNA feature is detected. This raises the question of what precise molecular signals provide a direct trigger for epigenetic silencing by HUSH, and how are H3K9me3-negative HUSH targets protected from it. We speculate that A-skew, the presence of cryptic termination sites, and the lack of introns—features commonly found in TEs and viruses—combine to promote the deployment of HUSH-mediated silencing. Interestingly, L1s are a major source of alternative polyadenylation and premature termination sites in the genome, and this has been in part attributed to their A-skew, a feature that is also important for HUSH-mediated silencing and slow elongation through L1 sequences.^12,44–46^ Moreover, A/T content rather than the presence of a canonical PAS is understood to control termination of non-coding RNAs.^43^

Although the HUSH complex was found to be vertebrate-specific, the function of WDR82 in transcription termination is highly conserved across evolution. The WDR82 homolog in yeast, called Swd2, is a member of the cleavage and polyadenylation factor (CPF) complex and interacts with the PP1/Dis2 phosphatase complex homologous to the mammalian PNUTS-PP1 to promote 3′ end transcript processing.^31,35,47–51^ Interestingly, although yeast lacks HUSH, an analogous process that couples CPF complex recruitment to non-canonical TTS with heterochromatin assembly has been recently described in fission yeast.^52^ Thus, connections between termination machinery and heterochromatin formation may represent an ancient mechanism that during vertebrate evolution has been exploited by HUSH.

The phenomenon of RNA-directed transcriptional gene silencing (TGS) has been described in many organisms including yeast, plants, and worms; however, the significance of TGS remains controversial in mammals.^8^ The majority of TGS mechanisms in model organisms rely on the production of small RNAs, in a manner dependent on the RNAi component Dicer and on the RNA-dependent RNA polymerase (RdRP) that amplifies the RNAi response by producing double-stranded RNA substrate for Dicer.^9^ These small RNAs are subsequently loaded onto the RITS complex to initiate gene silencing in an RNAPII-dependent manner.^9^ Mammals lack the RdRP activity, and the major mechanism of small RNA-dependent TGS convincingly established in the mammalian system is that involving piRNAs, a class of TE-derived small RNAs expressed selectively in the germline. It is therefore intriguing that structural parallels have been recently drawn between HUSH and the yeast RITS complex.^22^ However, we demonstrate here that HUSH silencing is Dicer-independent. Moreover, the recently described mechanism linking transcription termination and heterochromatin assembly in fission yeast is also RNAi-independent.^52^

Nonetheless, HUSH silencing is transcription-dependent and thus represents a novel TGS pathway uniquely present in vertebrates. At least two components of HUSH, PPHLN1,^12^ and MPP8 (this study) have been shown to bind RNAs at the sites of HUSH chromatin occupancy. However, the evidence that HUSH binding to chromatin is RNA-directed is currently lacking, and an alternative explanation for current observations is that the association of HUSH with RNA is simply a consequence of the proximity to the ongoing nascent transcription. Genomic sites at which RNAPII slows down could provide a window of opportunity for HUSH to effectively compete for the nascent RNA substrate with components of the RNA processing machinery rich in RNA-binding proteins. To what extent such interactions with RNA contribute to HUSH targeting or function remains to be established. We note potential parallels with HP1, a chromodomain protein which—similarly to MPP8—directly binds both H3K9me3-modified histones and RNA.^53,54^ In fission yeast, binding of HP1 to RNAs transcribed at the heterochromatin assembly sites weakens its association with H3K9me3, leading to the transcript release from heterochromatin and destruction via RNA decay pathway.^55^

Our study emphasizes the diversity of HUSH targets and also underscores the fact that many of the MPP8-bound genomic sites are independent of TASOR, a defining subunit of HUSH (Figure 7B). Therefore, caution needs to be taken when attributing MPP8-related phenotypes to HUSH. We further showed that the deletion of a non-coding gene desert, devoid of annotated regulatory elements, can result in profound effects on gene expression by triggering HUSH-dependent TGS. This observation highlights a heretofore unappreciated mechanism by which non-coding genomic rearrangements and deletions can impact gene function by the recognition of the resulting transcribed structural variant site as non-self DNA.

### Limitations of the study

Our observation that a HUSH target can switch from H3K9me3-negative to H3K9me3-positive in response to the deletion of the TTS is limited to the *Sox2* locus. It remains to be established if this observation is generalizable to other H3K9me3-negative HUSH targets. Furthermore, our data support a model whereby HUSH travels with RNAPII and associated machinery; however, the precise molecular cue for HUSH-mediated silencing and its relationship with termination or splicing remains to be identified.

## STAR★METHODS

### RESOURCE AVAILABILITY

#### Lead contact

Further information and requests for resources and reagents should be directed to and will be fulfilled by the lead contact, Joanna Wysocka (wysocka@stanford.edu).

#### Materials availability

Cell lines and plasmids generated by the authors are available upon request to other researchers

#### Data and code availability

- ChIP-seq, RNA-seq, PRO-seq, and CLIP-seq data have been deposited at GEO and are publicly available as of the date of publication under GEO: GSE208753. This paper analyzes existing, publicly available data; the accession numbers for the datasets are listed in the key resource table.
- This paper does not report original code.
- Any additional information required to reanalyze the data reported in this paper is available from the lead contact upon request.

### EXPERIMENTAL MODEL AND SUBJECT DETAILS

#### Culture of mouse embryonic stem cells

R1 mESCs were cultured as previously described.^66^ Briefly, male R1 cells were grown in monolayer on tissue culture plates pretreated with 7.5 ug/ml polyL-ornithine (Sigma) and 5 ug/ml laminine (BD) consecutively for 1 hour each in PBS. Cells were maintained in serum-free 2i + LIF media containing MEK inhibitor PD0325907 (0.8 uM), GSK3β inhibitor CHIR99021 (3.3 μM), and leukemia inhibitory factor (LIF).

### METHOD DETAILS

#### Endogenous tagging Sox2

Mouse embryonic stem cells were electroporated (Lonza nucleofection) per the manufacturers protocol with px458-mCherry^56^ carrying Cas9 and a single sgRNA targeting the C-terminus of Sox2 (TGCCCCTGTCGCACATGTGA) as well as two linearized pGEM-T (Promega) donors containing either GFP or BFP and 1 kb homology arms. GFP/BFP double positive cells were single- cell sorted by FACS and clonally selected.

#### Generating genomic rearrangement and TTS deletion in the Sox2 reporter

A heterozygous 100 kb deletion between Sox2 and its enhancer were generated by transient expression of Cas9 and a pair of sgRNAs (Upstream guide: GGCCGAATGATTAATAACG; downstream guide: AGCAGCTGAGCATCAAAGGG) in px458-mCherry^56^ with Lipofectamine 2000 (Thermo Fisher Scientific). Transfected cells were again sorted on mCherry as single cells and clonally selected. Clones were genotyped for heterozygous deletions by allele specific PCR primers both internal and external to the designed junction. Perturbation of the Sox2 TTS region was generated similarly using the following gRNAs: GACGCGCC GAGCGTTCTGCG and AAGAGGCGACACAGGCCTAG. Guide RNAs for the *Sox2–1kb* deletion and TTS deletion are listed in Table S4.

#### ChIP-seq

10–30 million sub-confluent cells were fixed on 10 cm plates with 1% methanol-free formaldehyde in PBS for 10 minutes at room temperature with nutation. Glycine was added dropwise to a final concentration of 125 mM to quench for an additional 5 minutes with nutation. Cross-linked cells were washed once with PBS before scraping on ice in cold PBS + 0.001% Triton X-100. Cell pellets were obtained by centrifugation at 1350 × g for 5 minutes at 4C, snap frozen in liquid nitrogen, and stored at −80C. On the day of the immunoprecipitation, pellets were thawed on ice for 30 minutes and resuspended in 5 mL LB1 (50 mM HEPES-KOH pH 7.5, 140 mM NaCl, 1 mM EDTA, 10% glycerol, 0.5% NP-40, 0.25% Triton X-100, with 1X Roche cOmplete Protease Inhibitor Cocktail) and rotated end over end at 4C for 5 minutes. Samples were centrifuged for 5 minutes at 4C and the pellet was resuspended in 5mL LB2 (10 mM Tris-HCl pH 8.0, 200 mM NaCl, 1 mM EDTA, 0.5 mM EGTA, with 1X Roche cOmplete Protease Inhibitor Cocktail) and again rotated end over end at 4C for 5 minutes. Samples were centrifuged for 5 minutes at 4C and the pellet was resuspended in 1 mL LB3 (10 mM Tris-HCl pH 8.0, 100 mM NaCl, 1 mM EDTA, 0.5 mM EGFTA, 0.1% sodium deoxycholate, 0.5% N-lauroylsarcosine, 1X Roche cOmplete Protease Inhibitor Cocktail, 1 mM PMSF) per ChIP (typically 5–10 million cells). Samples were sonicated in 1 mL Covaris AFA tubes for 5 minutes at peak power = 140, duty factor = 10, and cycles per burst = 20 in the E220 evolution Covaris.

After sonication, samples were spun at 14,000 × g for 10 minutes at 4C. Triton X-100 was added to the soluble supernatants to a final concentration of 1%. Fragmented chromatin was allocated for each ChIP and input sample (1%), and 7ug of the desired antibody was added to 1 mL of each ChIP. Inputs were stored at 4C and immunoprecipitations were rotated end over end at 4C overnight. Antibodies used in this paper include: MPP8 (Proteintech, 16796–1), GFP (Thermo Fisher Scientific, A-11122), H3K9me3 (Abcam, ab8898), WDR82 (Cell Signaling, #99715), and H3K4me3 (Abcam, ab8580).

100ul of magnetic protein G Dynabeads (Thermo Fisher) slurry was washed 4× 1 mL with 0.5% BSA in PBS for each IP. ChIPs were then added to pre-cleared protein G beads and rotated end over end at 4C for 4–6 hours. Chromatin-bound beads were then washed 5× 1mL with ice cold RIPA wash buffer (50 mM HEPES-KOH pH 7.5, 500 mM LiCl, 1 mM EDTA, 1% NP-40, 0.7% sodium deoxycholate), inverting the samples gently to mix. A final wash/buffer exchange was performed with 1 mL TE + 50 mM NaCl before aspirating the beads completely. Chromatin was eluted in Elution Buffer (50 mM Tris-HCl pH 8.0, 10 mM EDTA, 1% SDS) at 65C for 20 mins with 800 RPM agitation in a ThermoMixer (Eppendorf). Eluted chromatin and input samples were incubated overnight (>12 hours) at 65C to reverse formaldehyde crosslinking. Samples were diluted with one volume TE and digested with RNaseA (0.2 ug/ul final) for 2 hours at 37C and subsequently Proteinase K (0.2 ug/ul final) for 2 hours at 55C. DNA was cleaned up on ChIP DNA Clean & Concentrator columns (Zymo Research) and eluted in 50ul supplied DNA Elution Buffer.

For library preparation, ChIP and input DNA was quantified by Qubit dsDNA high sensitivity kit, and up to 50ng was used for combined end repair and A-tailing (NEBNext Ultra II) per the manufacturers protocol. Following Adaptor ligation and cleavage with the USER enzyme, samples were cleaned up on DNA Clean & Concentrator-5 columns (Zymo) and eluted in 11ul water. Size selection was performed on a 5% native PAGE gel. DNA in the range of 200–800 bp was excised and extracted overnight in crush-soak buffer (500 mM NaCl, 1 mM EDTA, 0.1% SDS) at 55C with agitation. Crush-soak slurry was filtered using Spin-X columns (Corning) and cleaned up on column. Libraries were amplified with the NEBNext Ultra II Q5 master mix and either included multiplex oligos or custom primers to add respective indices. Total cycle number was determined by qPCR. Amplified libraries were purified with sequential XP SPRI bead clean-up steps. Final quantification and QC was assessed by Bioanalyzer (Agilent) and libraries were pooled for sequencing on either Nextseq or NovaSeq (Illumina) platforms.

#### CRISPR pooled KO screen

Lentivirus harboring sgRNA libraries (10 guides per gene) was produced as previously described in 293FTs.^57^ Mouse embryonic stem cells harboring the endogenous Sox2 1kb reporter were transduced on 6-well plates with centrifugation and 8 ug/ml polybrene. Virus for each sub-library, concentrated from a 15 cm plate, was added to two, full 6-well plates (about 4M cells per well seeded the night before). Cells were centrifuged at 1000×g for two hours at 33C. Media was changed immediately after spinning and doxycycline was added at 2ug/ml. One day post transduction, infection efficiency and estimated viral titer was assessed by FACS analysis of mCherry positive cells on an LSR II flow cytometer (BD). Ideally this is around 30% for cells to receive one guide per cell. Puromycin was added at 1 ug/ml to select for sub-library integration. Selected cells were expanded in doxycycline for multiple passages to achieve 5×15cm plates per sub-library. Cells were released with Trypsin, washed with PBS, and resuspended in cold FACS buffer (2% FBS, 2 mM EDTA, 1% Sodium Azide in PBS). Cells were sorted at the Stanford Shared FACS Facility first for mCherry, indicating integration of the KO library, then for EGFP and TagBFP. Genomic DNA from sorted cells was harvested using the Gentra Puregene kit (Qiagen). Libraries for each sample were generated as previously described,^67^ and pooled for sequencing on the Nextseq (Illumina).

#### Co-IP

Protein G Dynabeads (Thermo Fisher) were washed three times with 1 mL cold GG Lysis Buffer (10 mM HEPES, 2 mM MgCl2, 10 mM KCl, 0.5% NP-40, 0.5 mM EDTA, 150 mM NaCl). Beads were conjugated overnight at 4C in bulk at a ratio of 50ul beads:5ug antibody in 1mL GG Lysis buffer rotated end over end. Mouse embryonic stem cells were washed once with PBS before lysing on-plate (1 mL per 10 cm plate) with ice cold GG lysis buffer + protease and phosphatase inhibitor cocktail tablets (Roche). Lysed cells were scraped from the plate and quickly moved to a tube on ice. Cells were homogenized on ice 15x with a pre-chilled 1 mL dounce. Lysate was centrifuged at 15,000 × g for 5 minutes at 4C to pellet insoluble material. Concentration of the soluble fraction was calculated using a BCA protein quantification kit (pierce) per the manufacturer’s protocol. Samples were diluted to 1–2 mg/ml with GG lysis buffer for IP. Pre-conjugated protein G beads were added to each IP at a ratio of 5 ug antibody per mg of protein. IP samples were rotated end over end at 4C for 4 hours. Post IP, beads were washed 4× 1mL with GG lysis buffer. Beads were spun briefly before returning to the magnet to remove all residual buffer. Beads were resuspended in 50 ul elution buffer (1x LDS, 0.1M DTT, GG lysis) and incubated at 95C for 10 mins. Eluate was harvested after returning beads to the magnet.

#### Total cell lysis and Western blot

Mouse embryonic stem cells were washed once with PBS before lysing on-plate in ice (1 mL per 10 cm plate) with ice cold RIPA buffer (50 mM Tris-HCl pH 7.4, 250 mM NaCl, 1 mM NP40, 0.5% sodium deoxycholate, and 0.1% SDS). Lysate was sonicated briefly with a Diagenode Bioruptor Plus for 4 cycles of 30 seconds on, 30 seconds off at 4C and centrifuged for 10 minutes at 14,000 × g at 4C. The soluble fraction was moved to a new tube and protein concentration was quantified using the Pierce BCA protein kit (Thermo Scientific). NuPAGE LDS Sample Buffer (Thermo Scientific) was added to a final concentration of 1x and DTT was added to a final concentration of 100 mM. Samples were incubated at 95C for 7 minutes before loading. Proteins were separated on a NuPAGE Bis-Tris gel run in MOPS SDS buffer (Invitrogen). Proteins were transferred to an Immobilon PVDF membrane (Millipore) in a tris-glycine transfer buffer containing 20% methanol at 350 mA for 1 hour at 4C. Membranes were blocked for 15 minutes with agitation in PBST (0.2% tween in PBS) containing 5% milk. Antibody dilutions were prepared in 5% milk PBST and added to blots in heat sealable strips. Blots were incubated with the respective primary antibody overnight at 4C with agitation. Membranes were washed four times with PBST for 5 minutes with agitation. Secondary antibodies were added at a dilution of 1:10,000 in 5% milk PBST, and membranes were incubated at room temperate for 1 hour with agitation. Membranes were washed extensively with PBST (at least 4 washes of 5 mins each). ECL prime (Amersham) was added briefly and removed prior to film exposure and development.

#### Endogenous tagging with AID-GFP-HA

A dsDNA gene block containing C-terminal ready nanoAID, sfGFP, and 3xHA sequences was purchased through IDT. The targeting construct was assembled in pGEM-T using a Gibson assembly master mix (NEB) and the EcoRV cut site. The assembly contained upstream and downstream homologous sequences to the c-terminus of the gene of interest as well as the AID-GFP-3xHA gene block. Cells were transfected with the targeting construct in combination with a single sgRNA and spCas9 expressed from the px458-mCherry plasmid (available on Addgene). Two days post transfection, GFP-positive single cells were sorted into fibronectin (5 ug/ml) coated 96-well plates with the help of the Stanford Shared FACS Facility. Clones were screened by PCR and/or Western blot for homozygous integration. Guide RNA sequences for endogenously tagging MPP8 and TASOR are available in Table S4.

#### rRNA-depleted RNA-seq library construction

Total RNA was extracted with TRIzol (1 ml per 10 cm plate). Chloroform (200 ul) was added to each sample, mixed by hand, and centrifuged for 10 minutes at 15,000×g. The aqueous phase was extracted and two volumes of 95% EtOH was added to each sample. Samples were vortexed briefly and added to an RNeasy spin column (Qiagen). DNase treatment, clean up, and elution was performed according to the manufacturer’s instructions. RNA was quantified on a nanodrop (Fisher Scientific) and 2 ug was used for ribosomal RNA depletion utilizing the Ribo Minus Eukaryote V2 kit (A15026; Invitrogen). rRNA-depleted RNA was quantified by RNA 6000 pico bioanalyzer (Agilent) and 20 ng was fragmented with the RNA fragmentation kit (Ambion). Briefly, RNA was pre-heated to 70C and 2 ul of 10x fragmentation buffer was spiked in for 2.5 mins at 70C. Next, 2ul of stop solution was added to quench the reaction on ice. Fragmented RNA was brought up to 100 ul with water along with 200 ul of RLT buffer (Qiagen) and 300 ul 95% EtOH. Samples were vortexed briefly before binding and cleaning up on an RNeasy column (Qiagen). RNA was eluted twice in 50ul water each for a total volume of 100 ul. RNA was lyophilized completely and resuspended in 5 ul water. Library construction and barcoding was carried out using the Ovation RNA-seq FFPE kit (Nugen). Post-amplification libraries were lyophilized, run on a native PAGE gel, and size selected to remove any free adaptor or primer. DNA was extracted from the PAGE gel overnight using the crush-soak method and cleaned on a DNA clean and conventrator-5 column (Zymo). Pooled libraries were denatured with NaOH and sequenced on the NextSeq 500 (Illumina) as a 2×75 run.

#### Mass spectrometry processing

Sample processing was performed as previously described.^68^ Specifically, protein samples were size-separated on bis-tris SDS-PAGE gels (Bio-Rad), and the gel was fixed and stained with the Colloidal Blue Staining Kit (Thermo Fisher Scientific) as per the manufacturer’s instructions. Each ChIRP-MS experiment (1 lane in the gel) was cut into 2 slices from the SDS-PAGE and prepared independently. Gel slices were prepared for mass spectrometry by rinsing sequentially in 200 μL HPLC-grade water, 100% Acetonitrile (ACN, ThermoFisher Scientific), 50 mM Ammonium Bicarbonate (AmBic). Samples were reduced by adding 200 μL of 5 mM DTT in 50 mM AmBic and incubating at 65°C for 30 min. The reduction buffer was discarded, and samples were cooled to room temperature. Alkylation was achieved by adding 200 μL of 25 mM iodoacetamide in 50 mM AmBic for 20 min at 25°C in the dark. The alkylation buffer was discarded, samples were rinsed once in 200 μL 50 mM AmBic, and then they were washed twice for 10 min each in 200 μL of freshly prepared 50% ACN in 50 mM AmBic. After each wash, the supernatant was discarded, and after all washes, samples were dried for 3 h using a SpeedVac. Once dry, proteins were digested by adding 100 ng of trypsin in 200 mL of 50 mM AmBic for 16 h at 37°C. Samples were subsequently acidified by adding formic acid to a final concentration of 1% and incubating at 37°C for 45 min. Finally, samples were desalted using C18 HyperSep Filter Plates with a 5–7 μL bed volume (ThermoFisher Scientific) following the manufacturer’s instructions. Samples were eluted twice in 100 μL 80% ACN in 0.2% formic acid, dried on a lyophilizer, and resuspended in 10 μL 0.1% formic acid for mass spectrometry analysis.

All samples were resuspended in 10 μL 0.2% formic acid in water and 4 μL were injected on column for each sample. Peptides were separated over a 50 cm EasySpray reversed phase LC column (75 μm inner diameter packed with 2 μm, 100 Å, PepMap C18 particles, Thermo Fisher Scientific). The mobile phases (A: water with 0.2% formic acid and B: acetonitrile with 0.2% formic acid) were driven and controlled by a Dionex Ultimate 3000 RPLCnano system (Thermo Fisher Scientific). An integrated loading pump was used to load peptides onto a trap column (Acclaim PepMap 100 C18, 5 um particles, 20 mm length, ThermoFisher) at 5 μL/min, which was put in line with the analytical column 6 min into the gradient for the total protein samples. Gradient elution was performed at 300 nL/min. The gradient increased from 0% to 5% B over the first 6 min of the analysis, followed by an increase from 5% to 25% B from 6 to 86 min, an increase from 25% to 90% B from 86 to 94 min, isocratic flow at 90% B from 94 to 102 min, and a re-equilibration at 0% for 18 min for a total analysis time of 120 min. Precursors were ionized using an EASY-Spray ionization source (ThermoFisher Scientific) source held at +2.2 kV compared to ground, and the column was held at 45°C. The inlet capillary temperature was held at 275°C, and the RF lens was held at 60%. Survey scans of peptide precursors were collected in the Orbitrap from 350–1350 Th with an AGC target of 1,000,000, a maximum injection time of 50 ms, and a resolution of 120,000 at 200 m/z. Monoisotopic precursor selection was enabled for peptide isotopic distributions, precursors of z = 2–5 were selected for data-dependent MS/MS scans for 2 s of cycle time, and dynamic exclusion was set to 45 s with a ± 10 ppm window set around the precursor monoisotope. An isolation window of 1 Th was used to select precursor ions with the quadrupole. MS/MS scans were collected using HCD at 30 normalized collision energy (nce) with an AGC target of 50,000 and a maximum injection time of 54 ms. Mass analysis was performed in the Orbitrap with a resolution of 30,000 at 200 m/z and an automatically determined mass range.

#### Infrared crosslinking and immunoprecipitation (irCLIP)

irCLIP was performed as previously reported.^69^ R1 mouse embryonic stem cells were grown as described above and protein-RNA interactions were fixed with UV crosslinking to a total of 0.35 J cm^−2^. Whole-cell lysates were generated in CLIP lysis buffer (50 mM HEPES, 200 mM NaCl, 1 mM EDTA, 10% glycerol, 0.1% NP-40, 0.2% Triton X-100 and 0.5% *N*-lauroylsarcosine) and briefly sonicated using a probe-tip Branson sonicator to solubilize the chromatin. Each experiment was normalized to the total protein amount, typically 1 mg, and partially digested with RNase A (Thermo Fisher Scientific, EN0531) for 10 min at 37 °C and then quenched on ice. Endogenous MPP8 protein was captured with protein G dynabeads pre-conjugated to MPP8 antibody (Proteintech; 16796–1) for 16 hours at 4 °C with rotation. The samples were sequentially washed (1 min each wash) at 25 °C in 1-mL volumes as follows: 2x high stringency buffer (15 mM Tris–HCl, pH 7.5, 5 mM EDTA, 2.5 mM EGTA, 1% Triton X-100, 1% sodium deoxycholate, 120 mM NaCl and 25 mM KCl), 1x high salt buffer (15 mM Tris–HCl, pH 7.5, 5 mM EDTA, 2.5 mM EGTA, 1% Triton X-100, 1% sodium deoxycholate and 1 M NaCl) and 2x NT2 buffer (50 mM Tris–HCl, pH 7.5, 150 mM NaCl, 1 mM MgCl_2_ and 0.05% NP-40). The RNA–protein complexes were dephosphorylated after the NT2 washes with T4 PNK (NEB) for 45 min in an Eppendorf Thermomixer at 37 °C, 15 s at 1,400 r.p.m., 90 s of rest in a 30 μL reaction, pH 6.5, containing 10 U T4 PNK, 0.1 μL SUPERase-IN (Thermo Fisher Scientific) and 6 mL PEG 400 (16.7% final). The dephosphorylated RNA–protein complexes were then rinsed once with NT2 buffer and 3′-end ligated overnight with T4 RNA ligase 1 (NEB) in an Eppendorf Thermomixer at 16 °C, 15 s at 1,400 r.p.m., 90 s of rest in a 60 μL reaction containing 10 U T4 RNA ligase, 1.5 pmol pre-adenylated-IR800–3′ biotin DNA-adaptor, 0.1 μL SUPERase-IN and 6 μl of PEG 400 (16.7% final). The following day, the samples were rinsed once with 500 mL NT2 buffer and resuspended in 30 μL of 20 mM dithiothreitol (DTT) and 1x LDS (Thermo Fisher Scientific) in NT2 buffer. The samples were heated to 75 °C for 10 min and the released RNA–protein complexes were separated by 4–12% bis-Tris SDS–PAGE (1.0 mm × 12 well) at 200 V for 45 min. The resolved ribonucleoprotein complexes were wet-transferred to nitrocellulose at 550 mA for 45 min at 4 °C.

The nitrocellulose membranes were imaged using an Odyssey CLx scanner (LiCor); the RBP–RNA complexes were excised using scalpels and the RNA was recovered by adding 0.1 mL Proteinase K reaction buffer (100 mM Tris, pH 7.5, 50 mM NaCl, 1 mM EDTA and 0.2% SDS) and 5 μL 20 mg/mL Proteinase K (Thermo Fisher Scientific). The proteins were digested for 60 min at 50 °C in an Eppendorf Thermomixer. Ligated RNA was captured with 5 μL of MyOne Streptavidin C1 Dynabeads (Thermo Fisher Scientific), which had been rinsed and suspended in 50 μL Biotin IP buffer (100 mM Tris, pH 7.5, 1 M NaCl, 1 mM EDTA and 0.1% Tween), and end-over-end rotation for 45 min at room temperature. The beads were placed on a magnet and washed three times sequentially with 100 μL Biotin IP buffer and once with 100 μL 1x PBS. cDNA synthesis occurred on the bead support by adding 12 μL water; 1 μL of 3 μM RT Primer and 1 μL of 10 mM dNTPs were added, heated to 70 °C for 5 min and then rapidly cooled to 4 °C. We then added cDNA master mix (4 μL 5×SuperScript IV (SSIV) buffer, 1 μL 100 mM DTT and 1 μL SuperScript IV; 6 μL total) to the annealed RNA and incubated for 30 min at 55 °C. After cDNA synthesis, the beads were placed on a magnet and washed twice sequentially with 100 μL Biotin IP buffer and once with 100 μL 1x PBS. The beads were resuspended in 10 μL cDNA elution buffer (8.25 μL water, 1 μL of 1 μM P3 short oligo and 0.75 μl of 50 mM MnCl_2_) and heated to 95 °C for 10 min. Subsequently, samples were cooled down at a rate of 0.1 °C s^−^ to 60 °C. Next, 5 μL circularization reaction buffer (3.3 μL water, 1.5 μL 10×Circligase II buffer and 0.5 μl Circligase II; Epicentre) was added. The cDNA was circularized for 2 h at 60 °C, followed by purification with 30 μL AMPure XP beads (Beckman Coulter) and 75 μL isopropanol. The samples were incubated for 20 min at 25 °C, washed twice with 100 μL 80% ethanol, air dried for 5 min and eluted in 14 μL water. Elution took place at 95 °C for 3 min and the samples were immediately transferred to a magnet. The eluted cDNA was transferred to a new PCR tube containing 15 μL 2×Phusion HF-PCR master mix (NEB), 0.5 μL of 30 μM P3/P6 PCR oligo mix and 0.5 μL 15×SYBR Green I (Thermo Fisher Scientific). Real-time qPCR was then performed as follows: 98 °C for 2 min; 15 cycles of 98 °C for 15 s, 65 °C for 30 s and 72 °C for 30 s, with data acquisition set to the 72 °C extension step. The PCR reactions were cleaned by adding 4.5 μL isopropanol, 54 μL AMPure XP beads and incubating for 10 min. The beads were washed once with 80% ethanol, dried for 5 min and eluted in 15 μL water. Illumina flow cell adaptors were added by adding 15 μL 2×Phusion HF-PCR master mix and 0.4 μL P3solexa/P6solexa oligo mix and amplified as follows: 98 °C for 2 min, followed by three cycles of 98 °C for 15 s, 65 °C for 30 s and 72 °C for 30 s. The final libraries were purified with the addition of 48 μL AMPure XP beads and incubation for 5 min. The beads were washed twice with 70% ethanol, dried for 5 min and eluted in 20 μL water. The libraries (1–2 μL) were quantitated using a HS-DNA Bioanalyzer. The samples were deep sequenced on the Illumina NextSeq machine (single-end, no index, high-output, 75-base-pair cycle run).

#### Single molecule fluorescence *in situ* hybridization

Mouse ESCs were hybridized with Stellaris RNA FISH Probe sets labeled with Quasar 570 (Biosearch Technologies, Inc.), following the manufacturer’s instructions available online at https://www.biosearchtech.com/support/resources/stellaris-protocols. Briefly, mESCs were cultured on fibronectin coated glass coverslips and fixed in 4% paraformaldehyde at room temperature for 10mins. mESC were then permeabilized overnight by 70% (vol./vol.) ethanol at 4°C. Coverslips were hybridized with 500 nM probes in hybridization buffer (10% Formamide, 10% 20x SSC, 10% Dextran sulfate, 1mg/mL Escherichia coli tRNA, 2mM Vanadyl ribonucleoside complex and 20ug/mL BSA) at 37 degree for 4 hours followed by washing and Hoechst staining. Custom Stellaris FISH Probe sets were designed against EGFP and TagBFP by utilizing the Stellaris^®^ RNA FISH Probe Designer v4.2 (Biosearch Technologies, Inc.) available online at https://www.biosearchtech.com/stellaris-designer. Probe sequences are available upon request. Z stack images were captured on wide-field microscope (Eclipse Ti, Nikon) equipped with a COMS camera (ORCA-Flash 4.0, Hamamatsu) and controlled by NIS-elements (Nikon). Objective with NA 1.4 and 60X magnification yielded an xy pixel-size of 107 nm. 21 Z-slices were recorded with a 200 nm step-size. GFP and BFP transcript numbers were estimated with FISH-quant.^70^

#### PRO-seq permeabilization

Cell permeabilization for PRO-seq was carried out under the advisement of the Nascent Transcriptomics core at Harvard Medical School, Boston, MA. Specifically, 10 cm plates of mESCs were washed once on-plate with PBS and released with Trypsin before quenching with ice cold DMEM + 10% FBS. Cells were counted and 15M cells were aliquoted per sample in 15 mL tubes.

#### PRO-seq library construction

Aliquots of frozen (−80°C) permeabilized cells were thawed on ice and pipetted gently to fully resuspend. Aliquots were removed and permeabilized cells were counted using a Luna II, Logos Biosystems instrument. For each sample, 1 million permeabilized cells were used for nuclear run-on, with 50,000 permeabilized *Drosophila* S2 cells added to each sample for normalization. Nuclear run-on assays and library preparation were performed essentially as previously described^71^ with modifications noted: 2X nuclear run-on buffer consisted of (10 mM Tris (pH 8), 10 mM MgCl2, 1 mM DTT, 300mM KCl, 20uM/ea biotin-11-NTPs (Perkin Elmer), 0.8U/uL SuperaseIN (Thermo), 1% sarkosyl). Run-on reactions were performed at 37°C. Adenylated 3’ adapter was prepared using the 5’ DNA adenylation kit (NEB) and ligated using T4 RNA ligase 2, truncated KQ (NEB, per manufacturer’s instructions with 15% PEG-8000 final) and incubated at 16°C overnight. 180uL of betaine blocking buffer (1.42g of betaine brought to 10mL with binding buffer supplemented to 0.6 uM blocking oligo (TCCGACGATCCCACGTTCCCGTGG/3InvdT/)) was mixed with ligations and incubated 5 min at 65°C and 2 min on ice prior to addition of streptavidin beads. After T4 polynucleotide kinase (NEB) treatment, beads were washed once each with high salt, low salt, and blocking oligo wash (0.25X T4 RNA ligase buffer (NEB), 0.3uM blocking oligo) solutions and resuspended in 5’ adapter mix (10 pmol 5’ adapter, 30 pmol blocking oligo, water). 5’ adapter ligation was per Reimer but with 15% PEG-8000 final. Eluted cDNA was amplified 5-cycles (NEBNext Ultra II Q5 master mix (NEB) with Illumina TruSeq PCR primers RP-1 and RPI-X) following the manufacturer’s suggested cycling protocol for library construction. A portion of preCR was serially diluted and for test amplification to determine optimal amplification of final libraries. Pooled libraries were sequenced using the Illumina NovaSeq platform.

#### ChIP-seq analysis

ChIP-seq fastq files were aligned to the mm10 assembly with BWA MEM. Peak calls were performed with macs3^60^ callpeak function with –broad option, mouse genome effective size and corresponding background reads. Visualization tracks were generated with bedtools genomecov (–bg –scale) with scaling factor being 10^6^ per number aligned reads and converted to bigWig with bedGraph-ToBigWig (Kent tools). BigWigs were visualized using the IGV browser.^59^ ChIP-seq interval overlap statistics were performed with bedtools fisher tools and visualized using R. A superset of ChIP peaks from various modifications was obtained with mean shift algorithm as described previously. ChIP coverage over intervals for scatter plots and statistical tests was obtained with bedtools coverage. H3K9me3+ regions were identified by performing DESeq2 analysis of the ChIP-seq counts in the intervals.

#### Heatmaps for ChIP-seq, RNA-seq, and PRO-seq

Heatmaps for ChIP-seq, RNA-seq and PRO-seq were generated by intersecting bam alignment files with intervals of interest using bedtools v2.25.0,^58^ followed by tabulation of the distances of the reads relative to the center of the interval and scaling to account for total aligned read numbers (10^6^ per number aligned). For CLIP data the bed file of RT-stops was used instead. Heatmaps were plotted using a custom R function. Aggregate plots were generated by averaging rows of the heatmap matrix. Similarly, bootstrap intervals were obtained by sampling with replicates the rows of the heatmap matrix, averaging the rows followed by determining 0.025 and 0.975 quantile. Heatmaps of the intersect of repetitive elements with HUSH peaks was obtained as described previously.^15^

#### CRISPR KO screen analysis

Initially, effect size and statistics for each sgRNA were determined by Cas9 high-Throughput maximum Likelihood Estimator (casTLE) as described previously,^63^ comparing the enrichment of on-target guides to that of non-targeting and safe-harbor guide RNAs in the library. To complement this approach, we used DESeq2^65^ to determine sgRNA enrichment and p-value for each gene.

#### irCLIP-seq analysis

RT stops were called utilizing the improved FAST-iCLIP pipeline as previously described.^69^ Briefly, duplicates were removed with fastx-collapser, 5’ barcodes and 3’ adaptors were trimmed, and STAR was used to align to the mm10 genome build.^61^

#### Mass spectrometry analysis

FASTA sequences of the human proteome (Uniprot: UP000005640) were used to map the peptides with MaxQuant^62^ with the following parameters: semi-specific cleavage specificity at the C-terminal site of R and K allowing for 2 missed cleavages. Mass tolerance was set at 12 ppm for MS1s, 0.4 for MS2s. Methionine oxidation, asparagine deamidation, and N-term acetylation were set as variable modifications. Cysteine carbamidomethylation was set as a fixed modification.

#### External datasets

External next generation sequencing data were downloaded from the Sequence Read Archive (SRA).

### QUANTIFICATION AND STATISTICAL ANALYSIS

Details regarding quantification and statistical analysis can be found in the corresponding figure legends and Experiment Details section of the STAR Methods.

## Supplementary Material

Table S2

Table S1

Table S3

Table S4

5

## Figures and Tables

**KEY RESOURCES TABLE:** 

REAGENT or RESOURCE	SOURCE	IDENTIFIER

Antibodies		

MPP8 – Rabbit polyclonal	Proteintech	Cat# 16796-1; RRID: AB_2266644
GFP – Rabbit polyclonal	Thermo Fisher Scientific	Cat# A11122; RRID: AB_221569
H3K9me3 – Rabbit polyclonal	Abcam	Cat# Ab8898; RRID: AB_306848
WDR82 – Rabbit monoclonal	Cell Signaling Technology	Cat# 99715; RRID: AB_2800319
H3K4me3 – Rabbit polyclonal	Abcam	Cat# ab8580; RRID: AB_306649
HRP conjugated mouse monoclonal anti-HSP90	Cell Signaling Technology	Cat# 79641S; RRID: AB_2799937
HA – Rabbit polyclonal	Abcam	Cat# ab9110; RRID: AB_307019
LINE-1 ORF1p-Rabbit monoclonal	Abcam	Cat# ab216324; RRID: AB_2921327

Bacterial and virus strains		

10-beta Competent *E. coli* (high efficiency)	NEB	C3019H
5-alpha Competent *E. coli*	NEB	C2987H

Chemicals, peptides, and recombinant proteins		

MEK inhibitor PD0325901	Selleck Chemicals	Cat# S1036
GSK3b inhibitor CHIR99021	Selleck Chemicals	Cat# S2924
polyL-ornithine	Sigma-Aldrich	Cat# P4638-100MG
Laminin	Life Technologies	Cat# 23017015
Fibronectin	ThermoFisher	Cat# FC01010MG
Indole-3-acetic acid sodium salt (auxin analog)	Sigma-Aldrich	Cat# 5148-2G
cOmplete, EDTA-free Protease Inhibitor Cocktail	Millipore Sigma	11873580001
PhosSTOP	Millipore Sigma	4906845001
SUPERaseIn	ThermoFisher	AM2694

Critical commercial assays		

Lipofectamine2000	ThermoFisher	Cat#11668019
NEBNext Ultra II DNA Library Prep Kit for Illumina	New England BioLabs	Cat# E7645S
TRIzol Reagent	Invitrogen	Cat# 15596018
NEBNext Multiplex Oligos for Illumina kit	New England BioLabs	Cat# E7335S
AMPure XP	Beckman Coulter	Cat# A63881
Dynabeads Protein G for Immunoprecipitation	Invitrogen	Cat# 10004D
Qubit dsDNA HS Assay Kit	Invitrogen	Cat# Q32854
Ovation RNA-seq FFPE	Nugen	discontinued
Unicersal Plus Total RNA-seq library preparation kit with NuQuant, mouse any-deplete	Tecan	Cat# 9157-24
RNeasy Plus Mini	Qiagen	Cat# 74134
DNA Clean & Concentrator-5	Zymo	Cat# D4014
HyperSep C18 Plates	ThermoFisher	60300-425

Deposited data		

ChIP-seq, RNA-seq, and PRO-seq	This paper	GEO: GSE208753
Western blot raw images	This paper	Mendeley DOI: 0.17632/v5y87syfcc.1

Experimental models: Cell lines		

R1 mESC WT	This paper	N/A
R1 mESC TASOR-AGH TIRI mCherry	This paper	N/A
R1 mESC TASOR-AGH TIR1-mCherry KO T1	This paper	N/A
R1 mESC TASOR-AGH TIR1 mCherry WDR82 KO T5	This paper	N/A
R1 mESC Sox2-1kb 1311 TRE Cas9 Blast	This paper	N/A
R1 mESC Sox2-1kb MPP8 KO M1 TRE Cas9 Blast	This paper	N/A
R1 mESC Sox2-1kb MPP8 KO M16 TRE Cas9 Blast	This paper	N/A
R1 mESC Sox2-100kb BG4	This paper	N/A
R1 mESC MPP8-AID-HA TIR1-mCherry	This paper	N/A
R1 mESC Sox2-1kb	This paper	N/A
R1 mESC CPSF3-AGH TIR1-mCherry	This paper	N/A
R1 mESC WDR82 KO W4	This paper	N/A
R1 mESC WDR82 KO W8	This paper	N/A
R1 mESC MPP8-AGH	This paper	N/A
R1 mESC Sox2-1kb DICER1 KO Clone 22	This paper	N/A
R1 mESC Sox2-1kb DICER1 KO Clone 23	This paper	N/A

Oligonucleotides		

Sox2 C-term endogenous tagging sgRNA: TGCCCCTGTCGCACATGTGA	This paper	N/A
Sox2-1kb deletion upstream sgRNA: GGCCGAATGATTAATAACG	This paper	N/A
Sox2-1kb deletion downstream sgRNA: AGCAGCTGAGCATCAAAGGG	This paper	N/A
Primers and sgRNA sequences, see Table S4	This paper	N/A

Recombinant DNA		

Super piggyBac Transposase expression vector	System Biosciences (SBI)	Cat# PB210PA-1
pGEM-T	Promega	Cat# A1360
Plasmid: pAS_pb_Ef1a-TIR1_Ubc-mCherry-Puro	This paper	Addgene Cat# 195567
Plasmid: pAS458-U6_CBA-mCherry	This paper	Addgene Cat# 195566
Plasmid: pbTRE3G-SpCas9-IRES-Blast	This paper	Addgene Cat# 195506
Plasmid: px458-mCherry	Gu et al.^56^	Addgene Cat# 161974
Plasmid library: Mouse CRISPR Deletion	Morgens et al.^57^	Addgene Cat# 1000000121
Library - Apoptosis and cancer		
Software and algorithms		
bedtools V2.25.0	Quinlan and Hall^58^	N/A
IGV	Thorvaldsdóttir et al.^59^	N/A
MACS3	Zhang et al.^60^	N/A
STAR	Dobin et al.^61^	N/A
MaxQuant	Cox and Mann^62^	N/A
CasTLE	Morgens et al.^63^	N/A
Bowtie 2	Langmead and Salzberg^64^	N/A
DESeq2	Michael Love^65^	N/A

Other		

NextSeq 500	Illumina	N/A
NovaSeq 6000	Illumina	
LightCycler 480	Roche	N/A
Bioanalyzer	Agilent	N/A
Amaxa 4D nucleofector	Lonza	N/A
Covaris sonicator E220	Covaris	N/A
LSRII Flow Cytometer	BD	N/A
FACSARIA II Flow Cytometer	BD	N/A

## Highlights

- CRISPR screen uncovers *de novo* silencing by HUSH upon structural gene rearrangement
- H3K9me3 separates distinct classes of HUSH targets
- HUSH accumulates at sites of high RNAPII occupancy including long exons and TTS
- Termination factors WDR82 and CPSF promote HUSH chromatin binding
