# Supplementary material for: Co-transcriptional genome surveillance by HUSH is coupled to termination machinery
Source: Mol Cell. Author manuscript; Available in PMC 2024 Mar 6. (PMC10915761; doi:10.1016/j.molcel.2023.04.014)
Supplement: 5 [file NIHMS1960707-supplement-5.pdf]

Molecular Cell, Volume *83*

Supplemental information

Co-transcriptional genome surveillance

by HUSH is coupled to termination machinery

Andrew L. Spencley, Shiran Bar, Tomek Swigut, Ryan A. Flynn, Cameron H. Lee, Liang-Fu Chen, Michael C. Bassik, and Joanna Wysocka

Supplementary Figure 1: Characterization of *Sox2-1kb* cell line. Related to Figures 1 and 2.

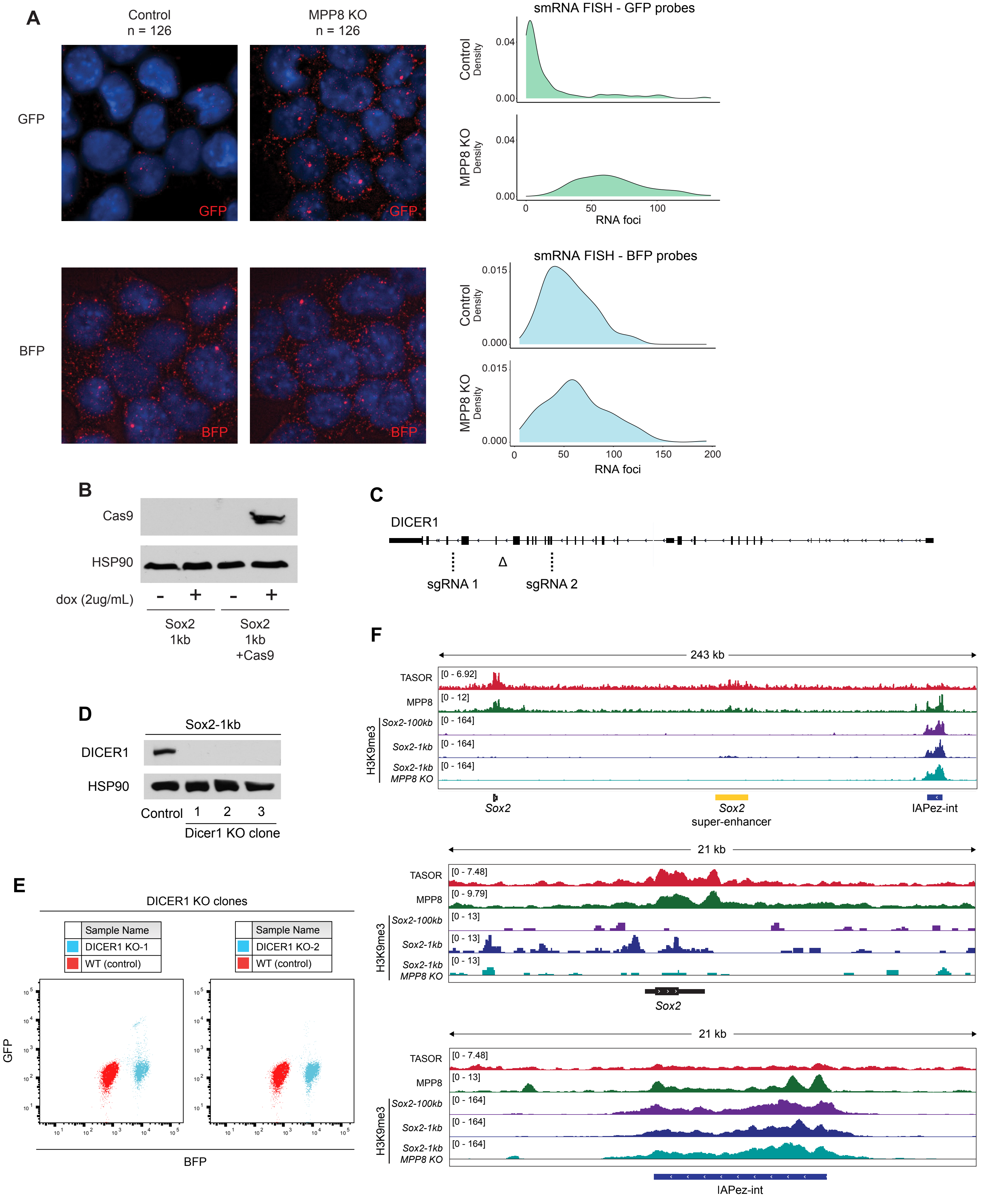

Supplementary Figure 2: Characterization of HUSH targets. Related to Figure 3.

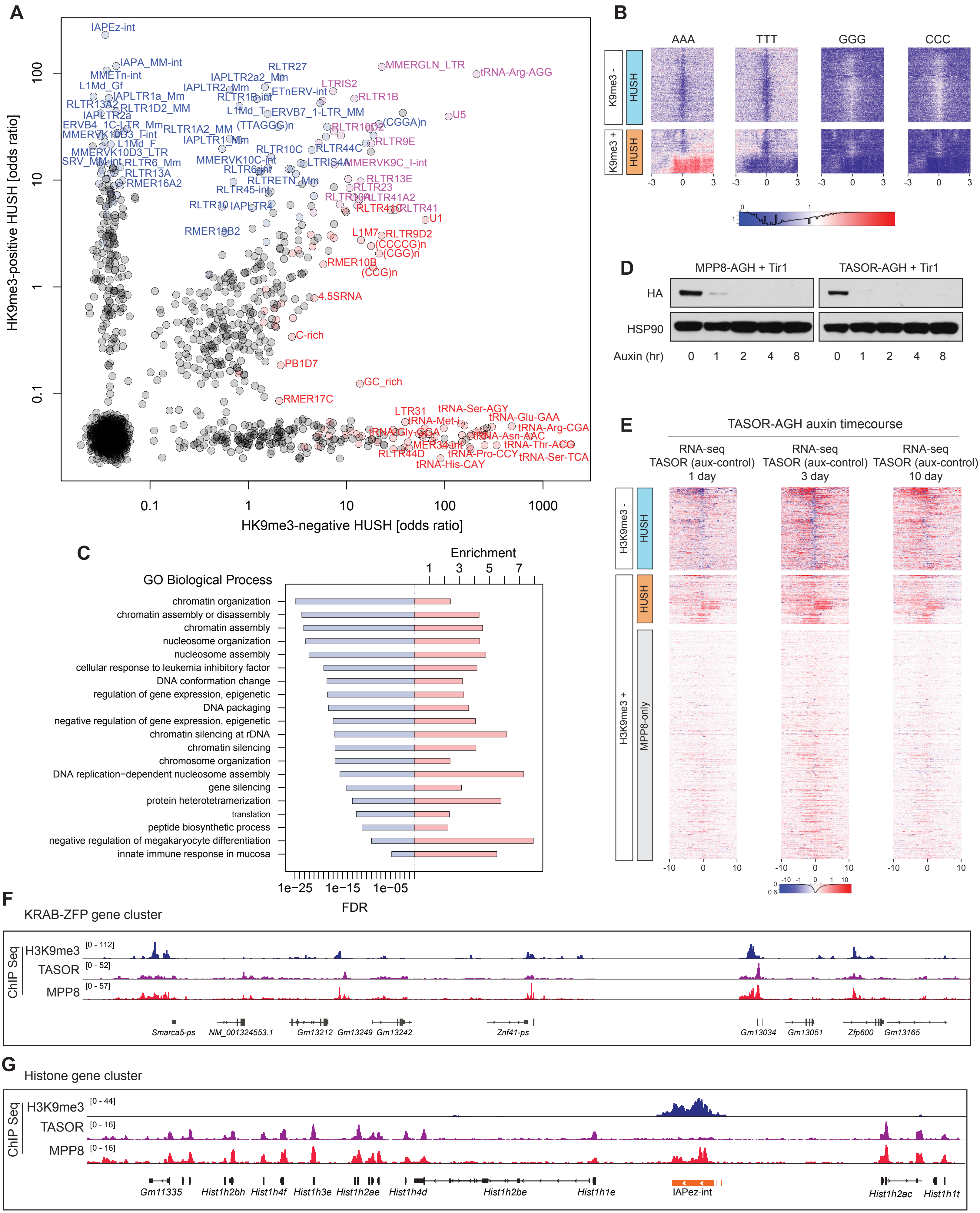

Supplementary Figure 3: MPP8 and TASOR irCLIP-seq. Related to Figure 4.

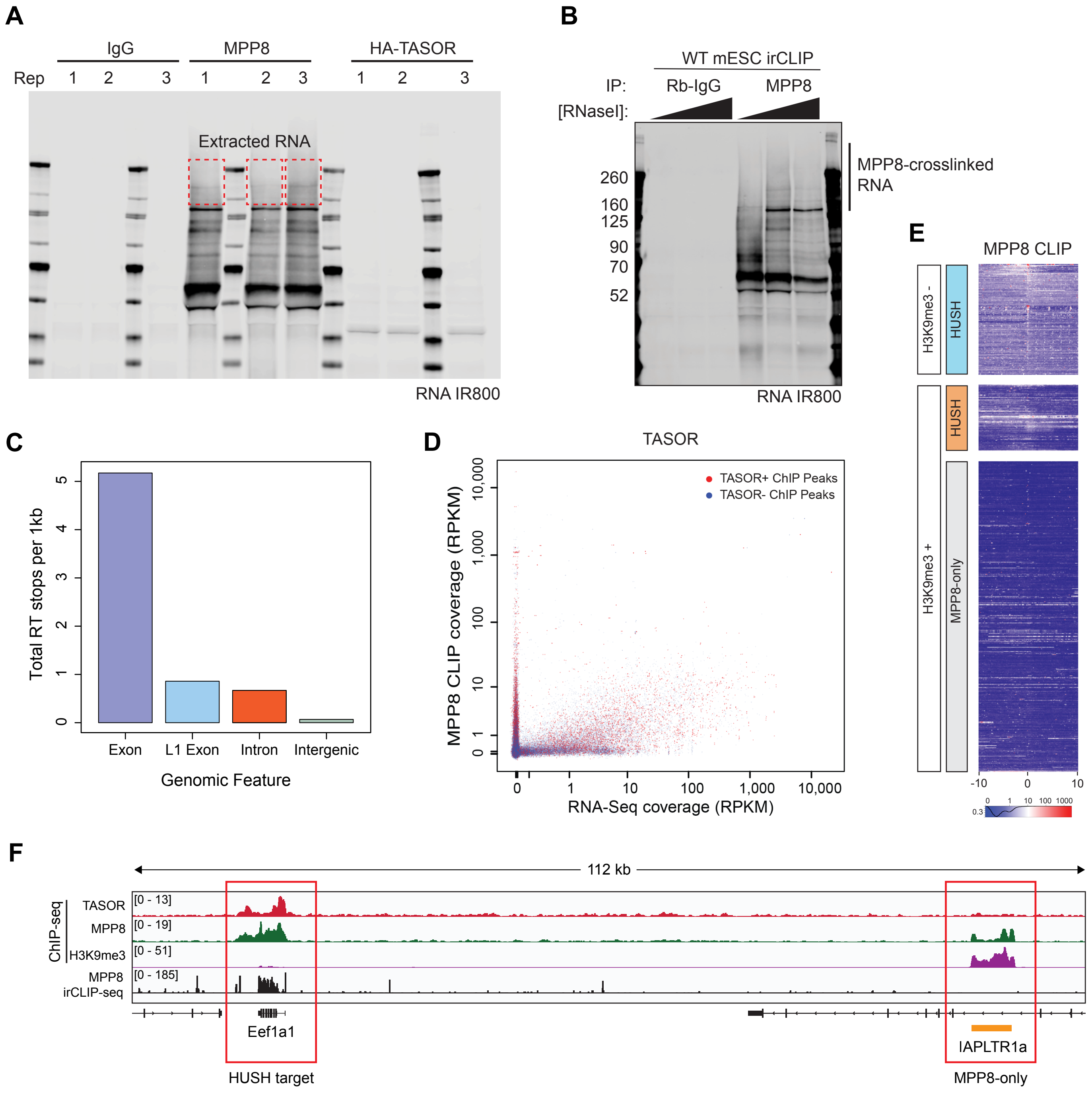

Supplementary Figure 4: HUSH, transcription, and WDR82. Related to Figure 5.

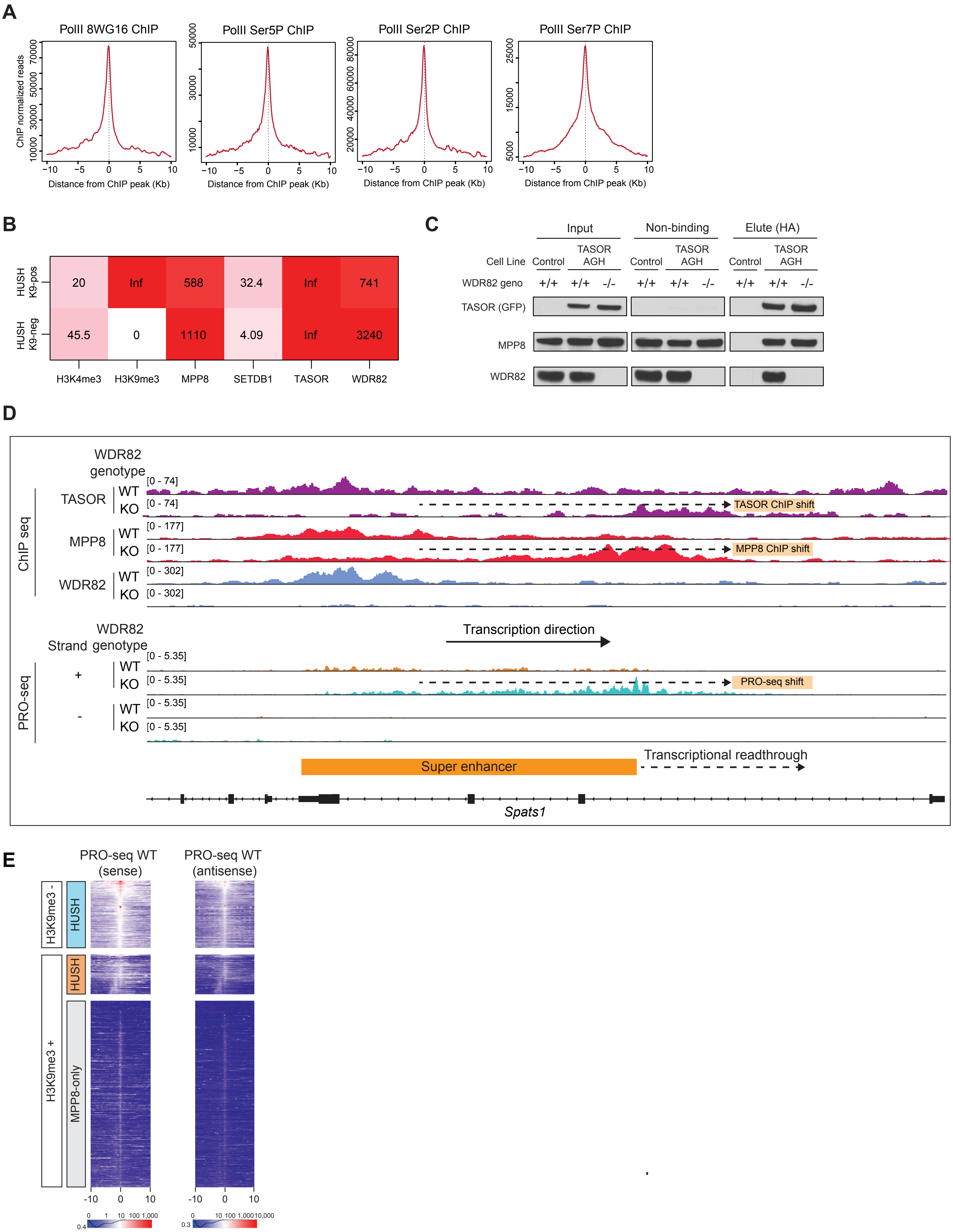

Supplementary Figure 5: ChIP-seq, PRO-seq browser tracks in WDR82 WT and KO. Related to Figure 5.

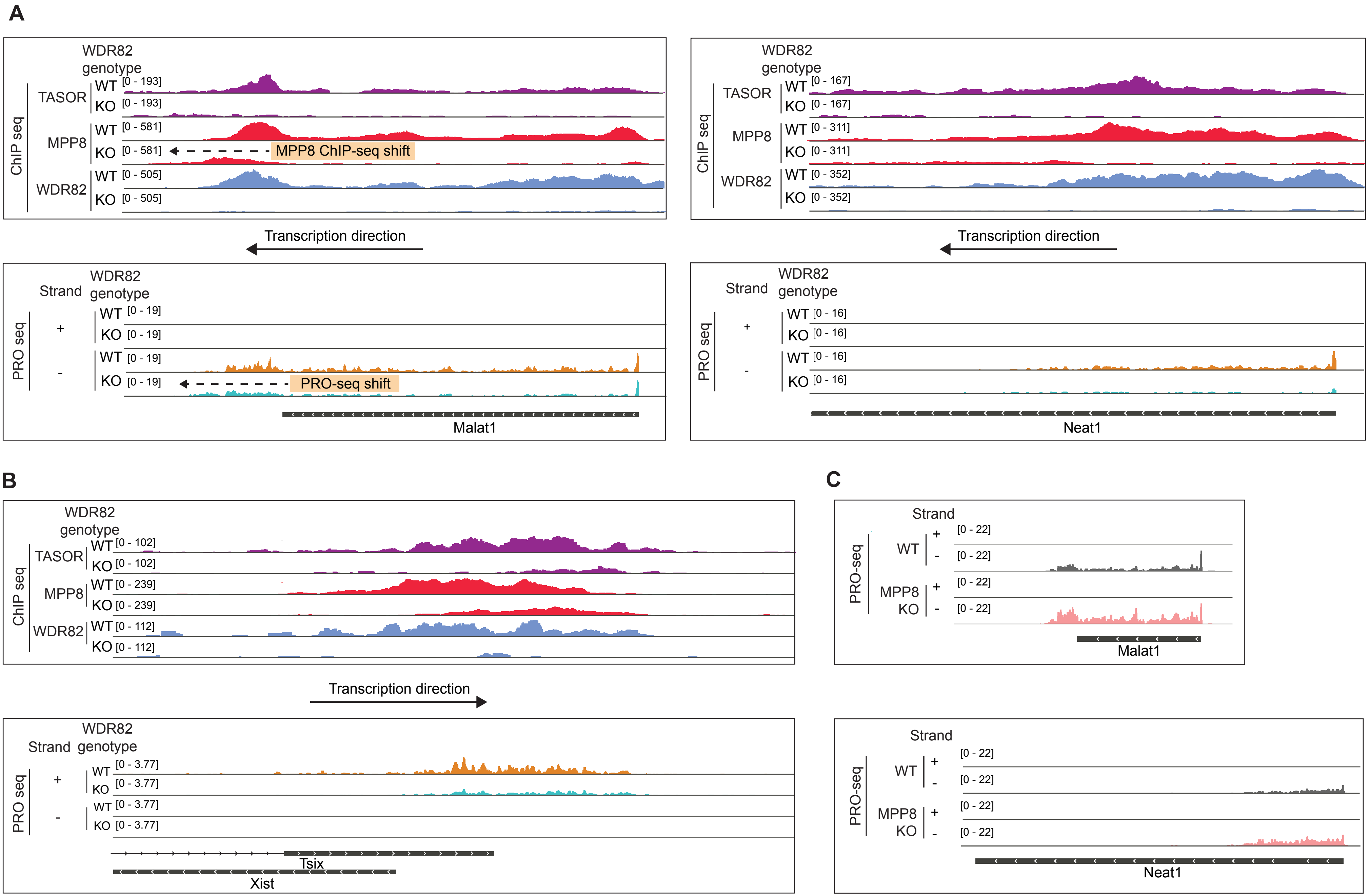

Supplementary Figure 6: WDR82 KO and TTS deletion in *Sox2-1kb* and *Sox2-100kb* cell lines. Related to Figure 6.

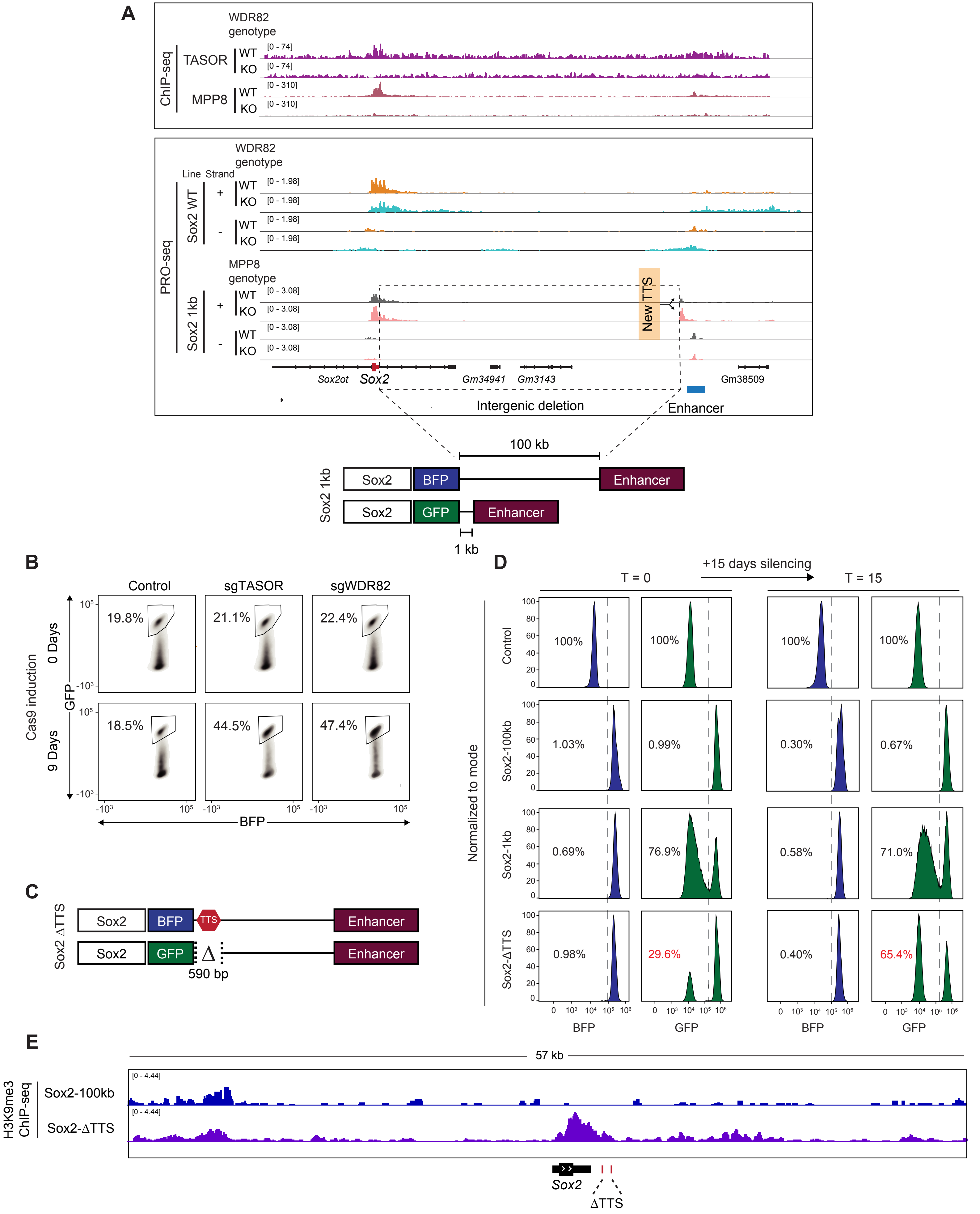

Supplementary Figure 7: WDR82 and MPP8 KO PRO-seq, CPSF3 degron. Related to Figure 6.

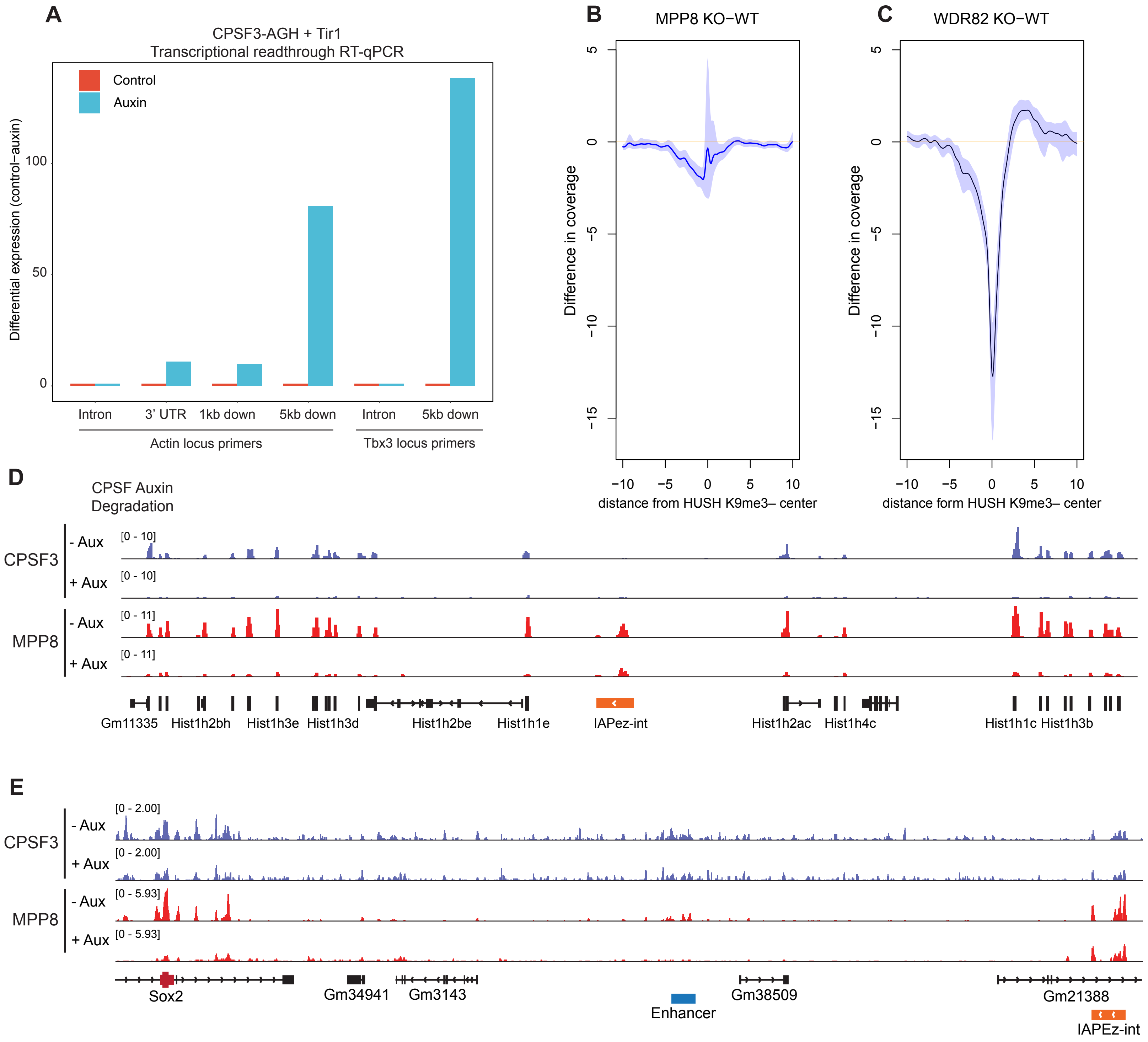

Supplementary figure legends:

Supplementary Figure 1: Characterization of the *Sox2-1kb* cell line. Related to Figure 1 and Figure 2.

(A) Single-molecule RNA fluorescence in-situ hybridization (FISH) with probes against GFP (top) or BFP (bottom) in *Sox2-1kb* control and MPP8 KO clone. Quantification of RNA puncta for GFP and BFP probes in both cell lines is indicated in histograms on the right.

(B) Western blot in *Sox2-1kb* cell lines, either with or without integration of doxycycline inducible Cas9 and indicated doxycycline treatment. Membranes were probed with antibodies raised against Cas9 and HSP90 as loading control.

(C) Schematic representation of the Dicer1 gene and location of sgRNAs used to generate KO clones.

(D) Western blot in Sox2-1kb control and Dicer1 KO clones. Membranes were probed with antibodies against DICER1 and HSP90 as loading control.

(E) FACS 2D plots of BFP (x-axis) and GFP (y-axis) in WT (red) and *Sox2-1kb* Dicer1 KO clones (blue).

(F) IGV browser tracks showing ChIP-seq for TASOR, MPP8, and H3K9me3 at the *Sox2* locus and downstream IAPez-int element. H3K9me3 is shown for *Sox2-100kb, Sox2-1kb,* and *Sox2-1kb MPP8 KO* cell lines to illustrate that H3K9me3 deposition is limited to *Sox2* and its super-enhancer in the *Sox2-1kb* deletion line. Zoomed in browser tracks for *Sox2,* and IAP element are also shown (bottom two panels).

Supplementary Figure 2: Characterization of HUSH targets. Related to Figure 3.

(A) Scatter plot of various repetitive elements separated by the odds ratio of their overlap with H3K9me3-negative (x-axis, red) or H3K9me3-positive (y-axis, blue) HUSH targets. Elements enriched in both groups of HUSH targets are shown in purple.

(B) Heatmaps showing enrichment of trinucleotide repeats at H3K9me3-negative (top) and H3K9me3-positive (bottom) HUSH ChIP-seq peaks. Heatmaps were centered and sorted as in Figure 3A. The x-axis represents distance from MPP8 ChIP peak in kb.

(C) GO term enrichment of biological processes determined by GREAT for H3K9me3-negative HUSH genomic targets.

(D) Western blot in MPP8-AGH (left) and TASOR-AGH (right) mESCs following treatment with auxin for the indicated number of hours. Membranes were probed with antibodies for HA and HSP90.

(E) RNA-seq coverage at HUSH and MPP8-only ChIP-seq peaks for TASOR degron time course in the TASOR-AGH + Tir1 cell line. Heatmaps are shown for 1-, 3-, and 10-day time points with enrichment of transcripts (red) or de-enrichment (blue) in auxin-treated cells relative to untreated control cells. Heatmaps were centered and sorted as in Figure 3A. The x-axis represents distance from MPP8 ChIP peak in kb.

(F) Browser tracks of TASOR, MPP8 and H3K9me3 ChIP-Seq in WT mESCs surrounding a large cluster of KRAB zinc-finger proteins (KZFPs).

(G) Same as (S2B), but at a large cluster of histone genes.

Supplementary Figure 3. MPP8 and TASOR irCLIP-seq. Related to Figure 4.

(A) irCLIP-PAGE gel indicating retrieved RNA from IgG, MPP8, and TASOR (HA) IPs in triplicate. RNA is visualized on LICOR with an IR800 dye. Extracted RNA from MPP8 irCLIP replicates is boxed in red.

(B) irCLIP-PAGE showing RNA signal for UV-crosslinked control (rabbit IgG) and MPP8 CLIP samples with increasing amounts of RNase I. Line indicates MPP8-bound RNA that collapses onto molecular weight of MPP8 protein with high RNase concentration and consequently shorter labelled RNA.

(C) Coverage of endogenous MPP8 irCLIP-seq at HUSH and MPP8-only sites centered and sorted as in Figure 3A. The x-axis represents distance from MPP8 ChIP-seq peak in kb.

(D) MPP8 irCLIP-seq RT stops as a percent of total (x-axis) mapped to a repeat-masked genome are shown for introns, exons, and intergenic regions (y-axis).

(E) Scatter plots showing all TASOR ChIP-seq peaks as a function of MPP8 CLIP coverage (y-axis) and RNA-seq coverage (x-axis). Marked in red are peaks overlapping TASOR peaks.

Supplementary Figure 4: HUSH, transcription, and WDR82. Related to Figure 5.

(A) Aggregate plots showing RNAPII ChIP-seq coverage (y-axis) relative to the distance from HUSH ChIP peak (x-axis) in WT mESCs. RNAPII ChIP with antibodies for various C-terminal domain (CTD) post translational modifications is shown.

(B) Heatmap displaying the odds ratio for the pairwise intersection of H3K9me3-negative and -positive HUSH peaks with various other ChIP-seq peaks as in Figure 5A.

(C) Western blot showing co-IP of MPP8 and WDR82 with TASOR in the TASOR-AGH tagged cell line and WDR82 WT and KO backgrounds. TASOR was immunoprecipitated with HA magnetic beads and peptide eluted. Input, non-binding, and eluted fractions are probed for TASOR (GFP), MPP8, and WDR82.

(D) Top: browser tracks of TASOR, MPP8, and WDR82 ChIP-seq in WT and WDR82 KO mESCs. Bottom: browser tracks of PRO-seq in WT (orange) and WDR82 KO (blue) mESCs for the positive (+) and negative (-) DNA strands. The dashed arrow marks the observed shifts in ChIP-seq and PRO-seq signals in WDR82 KO samples. The solid arrow indicates the direction of transcription as determined by PRO-seq.

(E) Heatmaps showing PRO-seq coverage in WT mESCs for sense (left) and antisense (right) strands, centered and sorted as in figure 3A. The x-axis represents distance from MPP8 ChIP peak in kb.

Supplementary Figure 5: ChIP-seq, PRO-seq browser tracks in WT and WDR82 KO. Related to Figure 5.

(A) and (B) Top: browser tracks of TASOR, MPP8, and WDR82 ChIP-seq in WT and WDR82 KO mESCs. Bottom: browser tracks of PRO-seq in WT (orange) and WDR82 KO (blue) mESCs for the positive (+) and negative (-) DNA strands.

(C) Browser tracks for PRO-seq in Sox2-1kb WT and Sox2-1kb MPP8 KO cells. The *Malat1* (top) and *Neat1* (bottom) loci are shown.

Supplementary Figure 6: WDR82 KO and TTS deletion in *Sox2-1kb* and *Sox2-100kb* cell lines. Related to Figure 6.

(A) Browser tracks showing ChIP-seq and PRO-seq at the *Sox2* locus. Top: TASOR and MPP8 ChIP-seq in WT and WDR82 KO mESCs. Bottom: PRO-seq in WT mESCs (orange), WDR82 KO (blue), *Sox2-1kb* control (grey), and MPP8 KO (pink) for the positive (+) and negative (-) DNA strands. The arrow marks the TTS that was generated following the intergenic deletion in the *Sox2-1kb* reporter, as schematically illustrated at the bottom.
(B) FACS 2D plots of BFP (x-axis) and GFP (y-axis) in *Sox2-1kb* reporter transduced with either non-targeting control sgRNAs (left) or sgRNAs targeting TASOR (center) or WDR82 (right). Shown are T0 and 9-day timepoints following induction of spCas9 expression.
(C) Schematic of endogenous *Sox2-100 kb* reporter showing heterozygous deletion of TTS in the GFP allele.
(D) FACS histograms displaying BFP- (blue) and GFP-positive (green) cells normalized to the mode in WT (control), Sox2-100 kb*,* Sox2-1kb (late passage), and *Sox2*-ΔTTS cell lines. Two timepoints are shown separated by 15 days to indicate the dynamics of silencing over time.
(E) Browser tracks of H3K9me3 ChIP-seq signal at the *Sox2* locus in *Sox2-100kb* (top) and *Sox2-ΔTTS* (bottom) cell lines. Position of the TTS deletion is indicated with red lines.

Supplementary Figure 7: WDR82 and MPP8 KO PRO-seq, CPSF3 degron. Related to Figure 6.
(A) RT-qPCR in CPSF3 depleted (blue) and control (red) mESCs, using intronic primers to capture nascent transcription or downstream primers to capture transcriptional readthrough in the Actin and Tbx3 loci.
(B) Aggregate plot of differential PRO-seq coverage. Plotted is the median and 95% CI of 1000x bootstrapped difference between MPP8 KO and WT. Ordinate represents relative distance to the polyA addition site overlapping H3K9me3-negative HUSH peaks in kb. The abscissa represents the difference in coverage (reads per billion per base per region).
(C) Browser tracks of CPSF3 and MPP8 ChIP-seq in control (no auxin) and CPSF3 depleted (12 hours after treating with auxin) mESCs surrounding a large cluster of histone genes.
(D) Same as (D), surrounding the *Sox2* locus.
